# Potentiation of the Pharmacological Effects of an *Aristolochia clematitis* L. Extract by Loading into Liposomes Facilitating Release to HaCaT Cells

**DOI:** 10.3390/pharmaceutics18010089

**Published:** 2026-01-10

**Authors:** Laura Grațiela Vicaș, Nicole Alina Marian, Diana Haj Ali, Narcis Duteanu, Paula Svera, Cristina Dehelean, Ana-Maria Vlase, Olimpia-Daniela Frenț, Ioana-Lavinia Dejeu, Rodica Anamaria Negrean, Răzvan Mihai Oros, Luminița Fritea, Andreea Smeu, Mariana Eugenia Mureșan

**Affiliations:** 1 Department of Pharmacy, Faculty of Medicine and Pharmacy, University of Oradea, No. 29 Nicolae Jiga Street, 410028 Oradea, Romania; lvicas@uoradea.ro (L.G.V.); daniela.olimpia@yahoo.com (O.-D.F.); ioana.dejeu@gmail.com (I.-L.D.); 2 Doctoral School of Biomedical Sciences, University of Oradea, No. 1 University Street, 410087 Oradea, Romania; mmuresan@uoradea.ro; 3 Department of Toxicology, Drug Industry, Management and Legislation, Faculty of Pharmacy, “Victor Babeș” University of Medicine and Pharmacy, 2 Eftimie Murgu Square, 300041 Timisoara, Romania; cadehelean@umft.ro (C.D.); andreea.smeu@umft.ro (A.S.); 4 Research Center for Pharmaco-Toxicological Evaluations, “Victor Babeș” University of Medicine and Pharmacy, 2 Eftimie Murgu Square, 300041 Timisoara, Romania; 5 Faculty of Industrial Chemistry and Environmental Engineering, Politehnica University of Timisoara, 2 Square Victoriei, 300006 Timisoara, Romania; narcis.duteanu@upt.ro; 6 National Institute for Research and Development in Electrochemistry and Condensed Matter, 144th Dr. A.P. Podeanu Street, 300569 Timisoara, Romania; paulasvera@gmail.com; 7 Department of Pharmaceutical Technology and Biopharmaceutics, Iuliu Hatieganu University of Medicine and Pharmacy, No. 12 Ion Creanga Street, 400010 Cluj-Napoca, Romania; gheldiu.ana@umfcluj.ro; 8 Department of Preclinical Disciplines, Faculty of Medicine and Pharmacy, University of Oradea, 10 Piata 1 Decembrie Street, 410073 Oradea, Romania; rodicanegrean@uoradea.ro (R.A.N.); mihaiorosrazvan@gmail.com (R.M.O.); lfritea@uoradea.ro (L.F.); 9 Clinical County Emergency Hospital Oradea, 65 Gh. Doja Street, 410169 Oradea, Romania

**Keywords:** *Aristolochia clematitis*, flavonoids, polyphenols, liposomes, antioxidants, antimicrobial activity, HaCaT cells

## Abstract

**Background:** *Aristolochia clematitis* L. (AC), a plant with diverse traditional uses, has gained increasing scientific interest due to its rich content of bioactive compounds such as flavonoids and polyphenols. However, its systemic use is limited by the presence of aristolochic acids, which are known for their nephrotoxic and carcinogenic potential. **Methods:** In this context, the present study investigates the therapeutic potential of *A. clematitis* extract by encapsulating it in liposomes with the aim of enhancing its topical efficacy. **Results:** The extract was characterized in terms of its flavonoid content (67.23 ± 0.33 mg QE/g DW (quercetin/dry plant material)) and polyphenols expressed as gallic acid equivalents (64.38 ± 0.16 mg GAE/g DW), as well as its antioxidant capacity using the reagents 1,1-diphenyl-2-picrylhydrazyl (DPPH − IC_50_ = 0.1619 mg/mL extract) and diammonium 2,2′-azino-bis(3-ethylbenzothiazoline-6-sulfonate) (ABTS − IC_50_ = 205.57 μg/mL extract). Four types of liposomes were synthesized (two loaded with extract and two empty), and their characterization was performed using Atomic Force Microscopy (AFM), Dynamic Light Scattering (DLS), Zeta Potential, polydispersity index, and in vitro release studies. **Conclusions:** The results demonstrated a high entrapment efficiency (over 82%), good stability over 30 days, and controlled release of flavonoids. Microbiological studies revealed relevant antimicrobial activity against *Staphylococcus aureus*, *Streptococcus pneumoniae*, *Escherichia coli*, and *Pseudomonas aeruginosa* strains. The evaluation on HaCaT skin-derived cells (at 10–100 µg/mL) proved that the samples displayed good overall tolerability, slightly decreasing cell viability (the most statistically significant being associated with AC treatment) and showing no structural, nuclear, or mitochondrial morphological changes.

## 1. Introduction

Plants have been used to treat various ailments since ancient times, and the biologically active natural compounds present as secondary metabolites exhibit multiple properties, such as antioxidant, antimicrobial, anti-inflammatory, analgesic, and antitumor effects, contributing significantly to health maintenance and disease prevention. The main classes of secondary metabolites identified in plants include polyphenols, alkaloids, tannins, saponins, lignins, glycosides, and terpenoids [1]. Among these compounds, according to current studies, polyphenols represent the most numerous and widespread group of biologically active natural compounds [2]. Flavonoids in plants can exhibit antioxidant effects and UV-protective properties, and they play an important role in regulating growth and differentiation processes, as well as in determining color and texture. They are therefore considered an essential component in a wide range of nutraceutical, pharmaceutical, medicinal, and cosmetic applications.

Beyond their general antioxidant properties, flavonoids have gained increasing attention as modulators of epidermal homeostasis, acting directly on keratinocytes, which represent the main functional cell population of the epidermis [3]. Recent mechanistic studies have demonstrated that flavonoids regulate key intracellular signaling pathways in keratinocytes, including NF-κB, MAPK/AP-1, JAK/STAT, and Nrf2/Keap1, thereby influencing oxidative stress responses, inflammatory mediator release, cell differentiation, and epidermal barrier integrity. Consequently, flavonoids are increasingly investigated as active agents for skin-targeted therapy in inflammatory, oxidative, and photo-induced skin disorders [4]. These compounds may protect the organism against degenerative conditions associated with oxidative stress, including cancer, osteoporosis, diabetes mellitus, asthma, neurodegenerative diseases, Parkinson’s disease, dementia, cardiovascular disorders, and inflammatory diseases [5,6].

*Aristolochia clematitis* L. (AC) has been used over the centuries for its traditionally perceived detoxifying, anti-inflammatory, and wound-healing properties, particularly when applied topically [7]. The herb and leaves of the plant are used in the form of poultices or compresses to treat rashes, pruritus, and skin edema. Moreover, the root, processed as a decoction, is used for the care of lesions caused by insect stings, purulent ulcers, eczema, wounds, inflammations, or gynecological conditions [8]. AC has attracted scientific interest due to its rich content of bioactive compounds, such as flavonoids, tannins, and alkaloids [9]. However, what has placed this plant at the center of attention in recent decades is also the reason its use has become extremely restricted: the presence of aristolochic acids, compounds that have been shown to be nephrotoxic and carcinogenic when administered systemically [10].

However, the effective topical application of flavonoid-rich plant extracts remains challenging due to their limited aqueous solubility, chemical instability, poor skin penetration, and rapid degradation under environmental stressors such as UV radiation and oxidation [11]. In this context, lipid-based nanoformulations have emerged as a strategic approach to improve flavonoid bioavailability at the epidermal level, enabling enhanced retention within the stratum corneum and viable epidermis while minimizing systemic exposure [12].

Nanocarrier-based delivery systems, particularly liposomes, have been extensively investigated as tools for modulating the biological behavior of bioactive compounds by altering cellular uptake pathways, biodistribution, and intracellular availability, rather than by chemically eliminating potentially toxic constituents [13,14,15]. This approach has garnered increasing attention in the context of complex plant extracts, which often contain both beneficial and harmful phytochemicals, enabling the selective exploitation of biological activity through controlled cellular exposure [16,17]. For AC, liposomal encapsulation represents a modern strategy to explore whether modulation of delivery and cellular interaction can influence antimicrobial activity and cellular responses.

About AC, exist a few patents address its application in dermatology. The scientific works use crude extracts or simple topical formulations for antioxidant, antimicrobial, or cosmetic purposes [18,19,20,21]. However, there is limited information on advanced delivery systems (liposomal formulations) that can improve the bioavailability, stability, and cellular uptake of active compounds. This justifies our study, which evaluates the biological activity of AC extract encapsulated in liposomes for potential topical applications.

Encapsulation in liposomes is an advantageous method that can increase the stability of bioactive compounds against environmental factors, reduce the number of side effects associated with the encapsulated substances, ensure controlled release of the active compound within the organism, and, due to their amphiphilic nature, allow the incorporation of both hydrophobic and hydrophilic bioactive molecules [22,23]. Recent studies have highlighted that liposomal encapsulation of flavonoids not only enhances their physicochemical stability but also modulates their cellular interaction with keratinocytes, influencing uptake, intracellular distribution, and sustained biological activity. In HaCaT-based in vitro models, flavonoid-loaded lipid vesicles have been shown to attenuate oxidative stress, suppress pro-inflammatory cytokine release, preserve mitochondrial function, and promote cytoprotective responses more efficiently than non-encapsulated counterparts. These findings support the concept that nanoencapsulation acts as a functional bridge between flavonoid composition and keratinocyte-specific biological responses [24].

This study aims to investigate the potential valorization of AC extract through liposomal encapsulation, enhancing the stability and local efficacy of its bioactive compounds, with particular emphasis on flavonoid-mediated modulation of keratinocyte viability and mitochondrial function. In the present study, the chemical characterization of the extract focused on total polyphenols and flavonoids. Due to the absence of a certified reference standard, aristolochic acids were not specifically identified or quantified by HPLC, and their presence cannot be excluded. Therefore, the antimicrobial activity and effects on HeLa cell viability should be interpreted as the result of the complex mixture of bioactive compounds present in the extract.

The main objective of the in vitro investigations was to assess the impact of the samples of interest (AC extract and obtaining liposomes) on HaCaT cells, an immortalized human keratinocyte cell line derived from human epithelial cells of adult skin. It was used as an experimental model to identify the biosafety and effects on the epidermal cells and to determine the potential suitability of these formulations for future dermatological applications. In this part, the samples were examined for 24 h in terms of cell viability using the MTT colorimetric test, cytotoxicity or cell membrane damage using the neutral red test, cell morphology in bright-field, analysis of the impact at the nuclear level, and analysis of the effects at the mitochondrial level.

## 2. Materials and Methods

Materials: The plant material used in this study comes from the spontaneous flora, carefully collected in June 2024, from unpolluted regions in the Oradea area (Bihor county, Romania). A specimen of the species was preserved in the herbarium of the Faculty of Medicine and Pharmacy Oradea, Romania, registered in the NYBG Steere Herbarium, UOP 05737. Only the leaves were selected for analysis.

Cholesterol lot STBC7280V, sodium cholate lot SLBZ1524, egg-yolk phosphatidylcholine lot BCBV3947, aluminum chloride (AlCl_3_), sodium nitrite (NaNO_2_), all sourced from Sigma-Aldrich Chemie GmbH, Steinheim, Germany. Phosphatidylserine lot SNRD8934, Sigma-Aldrich, Milan, Italy; methanol lot 2, Nordic Invest SRL, Cluj-Napoca, Romania; Folin-Ciocalteau’s reagent,1,1-diphenyl-2-pycrylhydrazyl (DPPH) was purchased from Sigma Aldrich (St. Louis, MO, USA). Quercetin, gallic acid and sodium carbonate from Triton-X from Decorias SRL, Rediu-Iași, Romania. Chloroform lot 19/07/25, Chempur, Piekary Śląskie, Poland. Phosphate buffer pH = 7.4, lot 3, Farmachim 10 SRL, Ploiești, Romania. ABTS (diammonium 2,2′-azino-bis(3-ethylbenzothiazoline-6-sulfonate) and potassium persulfate (K_2_S_2_O_8_) from Sigma Aldrich Chemie (Schnelldorf, Germany). Sodium hydroxide (NaOH) and methanol from Merck (Darmstadt, Germany). *Staphylococcus aureus* ATCC 25923 lot 01305403, *Streptococcus pneumoniae* ATCC 49169 lot 09511903, *Escherichia coli* ATCC 25922 lot 00208001, *Pseudomonas aeruginosa* ATCC 27853 lot 01007801, *Candida albicans* ATCC 10231 lot 0420621, Producer Tody Laboratories Int. SRL, București, Romania.

Culture media used: Mueller Hinton 2 LAB-AGAR, lot 51170160102BM, Mueller Hinton 2 LAB-AGAR + NAD + 5% KK, plate lot 00083310301NW (for *Streptococcus*) and Müller-Hinton 2 LAB AGAR supplemented with 2% glucose and methylene blue 0.5 mg/mL (for *Candida*), producer BIOMAXIMA, Cluj-Napoca, România.

For in vitro experiments, the MTT reagent (3-(4,5-dimethylthiazol-2-yl)-2,5-diphenyltetrazolium bromide) was purchased from Roche (Welwyn Garden City, UK), while the Hoechst 33342 nuclear stain and MitoTracker™ Red CMXRos were acquired from ThermoFisher Scientific (Waltham, MA, USA). Phosphate-buffered saline (PBS) and the penicillin-streptomycin antibiotic mixture were obtained from Sigma Aldrich, Merck KGaA (Darmstadt, Germany). Trypsin-EDTA, fetal bovine serum (FBS), and Dulbecco’s Modified Eagle Medium (DMEM) were supplied by PAN-Biotech GmbH (Aidenbach, Germany).

Equipment: Hot air oven (Pol-Eko, model SLW 115, Wodzislaw Śląski, A. Polok-Kowalska sp.k., Wodzisław Śląski, Poland); UV-VIS spectrophotometer T70+ (PG Instruments Ltd., Lutterworth, UK); rotary evaporator (Heidolph Hei-VAP Precision-Platinum3, Heidolph Instruments GmbH & Co. KG, Schwabach, Germany); analytical balance ABT 220-5DNM from Kern and Sohn GmbH (Balingen, Germany); ultrasonic bath Elmasonic S 100 H from Elma Schmidbauer GmbH (Singen, Germany); magnetic stirrer from Ecostris Dlab Scientific Co., Ltd. (Beijing, China); universal centrifuge 320 R from Hettich GmbH & Co. KG (Tuttlingen, Germany; Ratiopetta pipette 1000–5000 μL from Ratiolab GmbH (Dreieich, Germany); Franz diffusion cells (Microette-Hanson system, model 57-6AS9, Copley Scientific Ltd., Nottingham, UK); Scanning Probe Microscopy Platform (MultiView-2000 system, Nanonics Imaging Ltd., Jerusalem, Israel); DLS (Dynamic Light Scattering ZEN 3690) and Zetasizer Nano ZS (Malvern Panalytical, Malvern, UK). The Cytation 5 microplate reader, the Lionheart FX automated microscope, and the Gen5™ software for data acquisition and analysis (version 3.14) were obtained from BioTek Instruments Inc. (Winooski, VT, USA).

### 2.1. Preparation of the Hydroalcoholic Extract from *Aristolochia clematitis* L. (AC)

The plant material of AC was collected in July 2024 from the northwestern region of Romania, Bihor County. After harvesting, the leaves were separated, then washed and dried in the dark at 20 ± 2 °C for 14 days.

Obtaining the AC extract was carried out by maceration, according to the Romanian Pharmacopoeia, 10th Edition [25] and PhEur 11.8 [26]. A 70% methanol solution was used as the extraction solvent in a 1:10 (*w*/*v*) ratio, the proportion between plant material and solvent being 1:10. The resulting extract was used for subsequent determinations: HPLC analysis, total flavonoids, total polyphenols, and assessment of antioxidant activity by various methods.

### 2.2. HPLC-MS HPLC-MS Analysis

For the identification and quantitative determination of polyphenols and flavonoids, HPLC-MS was used, consisting of an Agilent Technologies 1100 Series HPLC system (Agilent, Santa Clara, CA, USA), equipped with a G1322A degasser, a G13311A binary gradient pump, a column thermostat, a G1313A autosampler, and a G1316A UV detector. This system was coupled to an Agilent 1100 mass spectrometer (LC/MSD Ion Trap VL). Data processing was performed using the Agilent ChemStation version B.01.03 and DataAnalysis software version 5.3 [27,28,29,30]. For this purpose, 5 µL of AC extract was injected onto a Zorbax SB-C18 reverse-phase analytical column (100 × 3.0 mm i.d., 3.5 µm particle size). The mobile phase consisted of a methanol:0.1% acetic acid mixture (*v*/*v*), with a flow rate of 1 mL/min and a working temperature of 48 °C. Compound detection was performed using UV at different wavelengths between 330 and 370 nm. MS detection was carried out using an electrospray ionization (ESI) source [27,28,29,30].

### 2.3. Total Flavonoid Content

Total flavonoid content was determined using a modified aluminum chloride colorimetric method [31]. As a calibration curve, the curve obtained by preparing standard solutions of 0.3, 0.6, 1.25, 2.5, and 3 mg quercetin equivalent/mL was used. The absorbance was measured at 510 nm using a UV-VIS T70+ spectrophotometer (PG Instruments Ltd., Lutterworth, UK). The calibration curve was obtained by plotting the absorbance of the standard solutions against the concentration of quercetin solutions, and it was found that the variation was linear within the concentration range of 0.3–3 mg quercetin equivalent/mL. This is represented by Equation (1):

(1) $$y\;=\;0.3112x\;+\;0.0527,\;R^{2}\;=\;0.9909$$

where y is the absorbance of the quercetin sample measured at 510 nm; x is the sample concentration, expressed in mg quercetin equivalents per mL (mg QE/mL). All determinations were performed in triplicate, and the results are expressed as mean value ± SD (standard deviation).

### 2.4. Determination of Total Polyphenol Content

For the determination of total polyphenol content, the most commonly used method is the Folin–Ciocalteu method, a spectrophotometric technique [32,33]. It is expressed as gallic acid equivalents per gram of plant material. The absorbance was measured with the UV-VIS T70+ spectrophotometer at a wavelength of 765 nm. The blank sample was prepared in the same way, except that no AC extract solution was added.

For determining the polyphenol concentration, a gallic acid calibration curve was used, employing solutions with concentrations between 0.01 and 0.15 mg GAE/mL. The calibration curve Equation (2) is:

y = 5.8542x + 0.1248, R^2^ = 0.9926 (2)

where y is the absorbance of the gallic acid solutions of different concentrations, measured at 765 nm; x is the concentration of the gallic acid solutions, expressed in mg gallic acid equivalents per mL (mg GAE/mL).

### 2.5. Evaluation of the Antioxidant Capacity of the Hydroalcoholic AC Extract

#### 2.5.1. DPPH Method (2,2-Diphenyl-1-picrylhydrazyl Method)

The in vitro evaluation of the antioxidant capacity of the plant extract can be determined using the DPPH technique, which employs the reagent 1,1-diphenyl-2-picrylhydrazyl. This is a spectrophotometric, simple, and rapid method used to assess the ability of compounds or plant extracts to neutralize free radicals by donating protons [34], through the scavenging of the DPPH radical. The absorbance was measured at 517 nm using a UV-VIS T70+ spectrophotometer [32]. For the blank sample, bidistilled water was used instead of the AC extract. All samples were prepared in triplicate. The determination of the DPPH inhibition percentage was carried out according to the following Equation (3):

(3) $$\%\;inhibition\;\mathrm{DPPH}=\frac{{A_{blanc}-A}_{sample}}{A_{blanc}}\cdot 100$$

where A_blank_ is the absorbance of the blank measured at 517 nm (t = 0 min); A_sample_ is the absorbance of the sample measured at 517 nm (t = 15 min).

#### 2.5.2. TEAC (Trolox Equivalent Antioxidant Capacity)—ABTS Method

For this method, the extract was reacted with an ABTS^+^ solution (2,29-azinobis-(3-ethylbenzothiazoline-6-sulfonic acid), and the absorbance was measured using a UV-VIS T70+ spectrophotometer at a wavelength of 734 nm. The results were interpreted using Trolox as the standard [33]. The blank sample was prepared in the same way, except that no AC extract was added. All determinations were performed in triplicate, and the results were expressed as mean value ± SD. The antioxidant capacity was expressed as the percentage of ABTS^+^ radical inhibition, and the results were obtained using Equation (4):

(4) $$\%\;inhibition\;\mathrm{ABTS}=\frac{{A_{blanc}-A}_{sample}}{A_{blanc}}\cdot 100$$

where A_blanc_ is the absorbance of the control (ABTS^+^ reagent dissolved in ethanol) measured at 734 nm (t = 0 min); A_sample_ is the absorbance of the sample (sample with ABTS^+^) measured at 734 nm (t = 6 min).

### 2.6. Preparation of Liposomes

For the preparation of liposomes, the lipid film hydration method was used [35]. The preparation of the extract for liposome formulation was carried out by evaporating the volatile fraction of the AC extract in an incubator until dry, at 30 °C.

For the lipid dispersion, 80 mg of phosphatidylcholine or phosphatidylserine, 20 mg of sodium cholate, and 2.5 mg of cholesterol were weighed and dissolved in 2 mL of chloroform, followed by stirring until complete dissolution. The volatile fraction of the solvent was removed using a rotary evaporator at 40 °C, with a rotation speed of 80 rpm and a pressure of 200 mBar, until a thin and uniform lipid film formed on the walls of the flask. Hydration of the lipid film was carried out with 2 mL of phosphate buffer solution (pH 7.4), in which the AC extract had been dissolved, followed by vigorous manual shaking. The dispersion was kept for 2 h at room temperature for stabilization and then mechanically shaken using a centrifuge under the following conditions: 40 °C, 500 rpm, for 20 min. Afterwards, the sample was sonicated for 30 min at 25 °C in an ultrasonic bath, and then stored in the refrigerator (4–8 °C) until analysis. Empty liposomes were prepared in the same manner, but without adding dried AC extract. The composition of the liposomes is presented in Table 1.

### 2.7. Determination of the Entrapment Efficiency of the Methanolic AC Extract

The entrapment efficiency of the methanolic AC extract was determined using the UV-VIS spectrophotometric method [36].

The separation of the obtained liposomes was performed after sonicating the liposomal solutions in an ultrasonic bath for 15 min at 30 °C. Then, 1 mL from each liposomal sample was transferred into a centrifuge and centrifuged at 5000 rpm, 5 °C, for 60 min. The pellet was washed with bidistilled water, after which 0.5% (*v*/*v*) Triton X was added to disrupt the vesicles and release the extract. To evaluate the amount of encapsulated extract, the total flavonoid content was determined using the colorimetric method described previously. Empty liposome samples were used as controls, and the entrapment efficiency, EE (%), was calculated using the equation:

EE (%) = TF/TFe × 100(5)

where TF is the amount of flavonoids encapsulated in the liposomes and TFe is the flavonoid content of the extract.

The entrapment efficiency (EE%) of the lipid vesicles loaded with AC extract, as well as the stability of the liposomes over time, were evaluated by performing the determinations at three different storage intervals (1, 15, and 30 days).

### 2.8. In Vitro Release of the AC Extract

The in vitro release study of the methanolic AC extract from the liposomes was carried out by determining the flavonoid content of the extract [35].

To evaluate the amount of flavonoids released from the liposomes, a six-cell Franz diffusion system was used, with a diffusion surface area of 1.767 cm^2^ and a receptor chamber volume of 6.5 mL. The receptor chamber of each diffusion cell was filled with freshly prepared phosphate buffer (pH 7.4) mixed with 30% ethanol. The synthetic membranes were made of polysulfone, with a diameter of 25 mm and a pore size of 0.45 μm—Tuffryn (R), PALL Life Sciences HT-450, lot T72556. They were prepared by hydrating them through immersion in phosphate buffer for 30 min prior to use, then mounted between the donor and receptor compartments of the Franz diffusion cells.

For the analysis, approximately 0.5 g of each type of liposome was placed in the donor compartment of the diffusion cells. The system was maintained at a temperature of 32 ± 1 °C, and the receptor medium was continuously stirred at 600 rpm. To determine the amount of released flavonoids, 0.5 mL samples from the receptor medium were withdrawn at different time intervals (30 min, 1, 2, 3, 4, 5, 6, 7, 8, 12, 24, and 48 h). The withdrawn volume was immediately replaced with fresh receptor medium to maintain a constant total volume of 6.5 mL.

### 2.9. Characterization of Liposomes

#### 2.9.1. Atomic Force Microscopy (AFM)

The analysis of the liposomes by AFM was carried out using the Scanning Probe Microscopy Platform (MultiView-2000 system) in intermittent mode, under ambient conditions (20 ± 2 °C). For the morphological analysis of the liposomes, a scanner equipped with a silicon probe coated with chromium, with a tip radius of 20 nm, operating at a resonance frequency of 30–40 kHz, was used. The liposomes were ultrasonicated for one hour at room temperature, then 0.2 mL was dropped onto a glass device support and left to dry at 20 ± 2 °C for one week [35]. The measurements were also performed after 30 days.

#### 2.9.2. Dynamic Light Scattering (DLS)

The characterization of liposome size and surface charge potential was performed using DLS (ZEN 3690) and the Zetasizer Nano ZS instrument (Malvern Panalytical, UK).

A volume of 2 mL of the liposomal solution was sonicated for 5 min and then transferred into a polystyrene cuvette with a 1 cm optical path, and measurements were performed for each sample. The results were presented according to the intensity and volume distribution of particle diameter (d.nm). The volume distribution was considered in order to compare the three possible size levels that liposomes may reach: very small vesicles, large vesicles, or flocculated structures [37,38,39].

The Zeta Potential was determined to assess the stability of the liposomal emulsion, using disposable folded capillary cells [35]. All measurements were performed in triplicate.

### 2.10. Microbiologic Analysis

The antimicrobial activity of AC extract and synthesized liposomes was evaluated against a representative panel of microorganisms, including Gram-positive bacteria (*S. aureus*, *S. pneumoniae*), Gram-negative bacteria (*E. coli*, *P. aeruginosa*), and yeast (*C. albicans*). This selection was made to cover a wide range of microbial cell types that differ in cell wall structure, metabolism, and susceptibility, allowing the evaluation of broad-spectrum antimicrobial potential.

The antimicrobial activity of AC was evaluated using the disk diffusion method, following standard methodology [40]. To determine the antimicrobial activity of the AC extract and the four types of liposomal solutions, the disk diffusion method was used on culture media poured uniformly in a thin 4 mm layer, pH 7.4. The following reference strains were used: *S. aureus* ATCC 25923 (lot 01305403), *S. pneumoniae* ATCC 49169 (lot 09511903), *E. coli* ATCC 25922 (lot 00208001), *P. aeruginosa* ATCC 27853 (lot 01007801), and *C. albicans* ATCC 10231 (lot 0420621), supplied by Tody Laboratories Int. SRL, Bucharest, Romania. The culture media used were: Mueller Hinton 2 LAB-AGAR (lot 51170160102BM), Mueller Hinton 2 LAB-AGAR + NAD + 5% sheep blood (lot 00083310301NW) for *S. pneumoniae*, and Mueller Hinton 2 LAB-AGAR supplemented with 2% glucose and methylene blue 0.5 mg/mL for *C. albicans*, produced by BIOMAXIMA, Cluj-Napoca, Romania.

The inocula were prepared in nutrient broth, with their density adjusted to a 0.5 McFarland turbidity standard for disk diffusion. The inoculated plates were incubated at 37 °C for 24 h, and turbidity was measured using a DEN 1 Biosan nephelometer (Biosan, Riga, Latvia). On each filter paper disk, 4 mm in diameter, 15 µg of the extract solution and of the liposomal solutions were applied, after which the disks were placed on the seeded plates. Empty liposome dispersions, without AC extract, were used as references. After 24 h of incubation at 37 °C, the diameters of the inhibition zones were measured, and the results were expressed in millimeters. Each test was performed in triplicate, and the values are presented as mean results.

### 2.11. Cell Culture Protocol

Cell Culture Conditions: The immortalized human keratinocyte cell line—HaCaT (300493) was sourced from CLS (Eppelheim, Germany). Cells were cultured according to the manufacturer’s recommendations, as follows: in Dulbecco’s Modified Eagle Medium (DMEM) supplemented with 10% fetal bovine serum (FBS) and 1% antibiotics (penicillin 100 U/mL and streptomycin 100 µg/mL), and incubated at 37 °C and 5% CO_2_.

Experimental Design: HaCaT cells (Passage 31) were treated with various concentrations of AC, PCA, PCE, PSA, and PSE (10, 25, 50, 75, and 100 µg/mL) for 24 h. The stock solutions were prepared in DMSO. The final concentration of DMSO in the samples did not exceed 0.5% (*v*/*v*).

#### 2.11.1. Cell Viability Assessment—The MTT Test

Cell viability was assessed using the MTT assay (3-(4,5-dimethylthiazol-2-yl)-2,5-diphenyltetrazolium bromide. The HaCaT cells were cultivated in flat-bottom 96-well plates at a density of 1 × 10^4^ cells/well, and subsequently treated for 24 h with AC, PCA, PCE, PSA, and PSE at concentrations of 10, 25, 50, 75, and 100 µg/mL. At the end of the treatment, the culture medium was replaced with fresh medium, followed by the addition of 10 µL of kit 1 to each well. The plates were then incubated for 3 h at 37 °C and 5% CO_2_. Subsequently, 100 µL of kit 2 was added to each well, and the plates were incubated at room temperature for 30 min. Finally, absorbance was measured at 570 nm and 630 nm using the Cytation 5 microplate reader.

#### 2.11.2. Neutral Red Staining

HaCaT cells cultured in DMEM supplemented with 10% FBS were seeded into 96-well flat-bottom plates at a density of 1 × 10^4^ cells/well and treated with AC, PCA, PCE, PSA, and PSE at concentrations of 10, 25, 50, 75, and 100 µg/mL. After the treatment period, the culture medium was replaced with 100 µL/well neutral red solution prepared in DMEM, containing the dye at 40 µg/mL to allow intracellular accumulation and the plates were incubated for 2 h at 37 °C and 5% CO_2_. The cells were then rinsed with PBS (150 µL/well) and imaged in brightfield using a Lionheart FX automated microscope. Dye release was carried out using 150 µL/well destaining mixture composed of approximately 50% ethanol, 49% ultrapure water, and 1% glacial acetic acid. The resulting extract was subsequently measured at 540 nm with a Cytation 5 plate reader to quantify neutral red retention. The experimental procedures were conducted in accordance with Repetto et al. [41].

#### 2.11.3. Cell Morphology Evaluation

To evaluate the effects of AC, PCA, PCE, PSA, and PSE at concentrations of 10, 25, 50, 75, and 100 µg/mL on cellular morphology, HaCaT cells were cultivated at a density of 1 × 10^4^ cells/well in 96-well plates. Morphological changes were examined by capturing images under brightfield illumination at 20× magnification using the Lionheart FX automated microscope. Image processing was performed with Gen5 Microplate Data Collection software, version 3.14 (BioTek Instruments Inc., Winooski, VT, USA).

#### 2.11.4. MitoTracker Red CMXRos—Mitochondrial and Hoechst 33342—Nuclear Immunofluorescence Stainings

To evaluate nuclear and mitochondrial alterations induced by the samples of interest in HaCaT cells, Hoechst 33342 and MitoTracker Red CMXRos immunofluorescence stainings were performed.

First, HaCaT cells were cultured in 12-well plates at 1 × 10^5^/well, and allowed to adhere until optimal confluence was achieved. The cells were then treated for 24 h with AC, PCA, PCE, PSA, and PSE at concentrations of 10, 25, 50, 75, and 100 µg/mL. The MitoTracker stock solution (1 mM in DMSO) was diluted in the complete culture medium to obtain a final concentration of 300 nM. The cells were then incubated with the staining solution for approximately 30–45 min under standard culture conditions, after which they were thoroughly washed with PBS to eliminate any residual dye. Following MitoTracker labeling, the cells were fixed in 4% paraformaldehyde for 10 min at room temperature to preserve cellular structures, and subsequently washed again with PBS.

To assess the nuclear changes triggered by AC, PCA, PCE, PSA, and PSE, Hoechst 33342 staining was carried out. Thus, a Hoechst 33342 working solution (prepared at a 1:2000 dilution in PBS) was added to each well, and plates were incubated for 5–10 min in the dark. After incubation, the dye was removed and the wells were rinsed three times with PBS. Fluorescence images were then acquired using a Lionheart FX automated microscope at 20× magnification, and the resulting data were processed with the Gen5™ Microplate Data Collection and Analysis Software (version 3.14, BioTek Instruments Inc., Winooski, VT, USA).

### 2.12. Statistical Analysis

The results are expressed as mean ± standard deviation (SD). Statistical analysis was conducted using one-way ANOVA and Dunnett’s multiple comparisons test. The analysis was performed using GraphPad Prism version 10.2.3 (GraphPad Software, San Diego, CA, USA; www.graphpad.com). The statistically significant were marked with “*”: * *p* < 0.05; ** *p* < 0.01; *** *p* < 0.001; **** *p* < 0.0001.

## 3. Results and Discussions

### 3.1. HPLC-MS Analysis

The compounds detected by mass spectrometry (MS) in the samples were compared with the specific mass spectra of each polyphenol recorded in a reference library. The compounds identified based on MS spectra were further quantified using UV spectrophotometry. For quantitative determination, the external standard method was applied, using calibration curves ranging from 0.5 to 50 µg/mL, with good linearity (R^2^ > 0.999). The limit of quantification for this method was 0.5 µg/mL, and the limit of detection was 0.1 µg/mL [42,43,44]. Only qualitatively, gentisic acid and chlorogenic acid were identified. Quantitatively, four polyphenolic compounds (1–4) (Supplementary Material) and four phenolic acids (5–8) were identified in the AC extract, with their concentrations expressed in μg/mL of extract (Table 2).

Isoquercitrin, also known as isoquercitine, is a flavonoid and the 3-O-glucoside of quercetin, and can be isolated from various plant species. The IUPAC name is 3-(β-D-glucopyranosyloxy)-3′,4′,5,7-tetrahydroxyflavone. In the AC extract, this compound was found in the highest amount, 35.837 μg/mL extract. Scientific literature reports several pharmacological activities for this compound, including antioxidant potential [45], reduced oxidative stress [46], anti-inflammatory activity [47], anti-cancer [48], antihypertensive and antidiabetic effect [49,50] and health benefits etc. [51].

Other flavonoid compounds identified in the AC extract, but in lower concentrations, include ferulic acid (extract 12.945 ± 0.388 μg/mL), which is known for its beneficial antioxidant activity [52], anticancer activity [53], anti-inflammatory activity [54]. Rutoside is also identified in low concentrations (8.840 ± 0.531 μg/mL), beneficial for its antioxidant, anti-inflammatory, anticancer, antidiabetic, neuroprotective, antiallergic, and antihypertensive activities, among others [55,56,57].

The high content of isoquercitrin, which is a glycosylated flavonoid, is consistent with the antioxidant capacity revealed by the DPPH and ABTS tests, which reflect the ability of the extract to neutralize free radicals through electron and hydrogen atom transfer mechanisms.

The IC_50_ values obtained by these methods highlight both the ability of the compounds to capture free radicals from a chemical system and their potential to participate in biological processes associated with redox homeostasis.

The phytochemical profile revealed by HPLC-MS may contribute to the manifestation of antimicrobial effects, observed in agar diffusion tests. The zones of inhibition obtained against the Gram-positive and Gram-negative strains studied are consistent with the antimicrobial mechanisms reported for flavonoids and phenolic acids, which include disruption of the integrity of the microbial membrane, interference with intracellular enzyme systems and induction of an oxidative imbalance at the bacterial level. The reduced sensitivity observed in the case of *Pseudomonas aeruginosa* is compatible with its poorly permeable external cell structure, which limits the penetration of phenolic compounds [58].

In contrast, the absence of detectable antifungal activity against *Candida albicans* ATCC 90029 suggests a predominantly antibacterial selectivity of the AC extract under the experimental conditions used.

**Figure 1:**
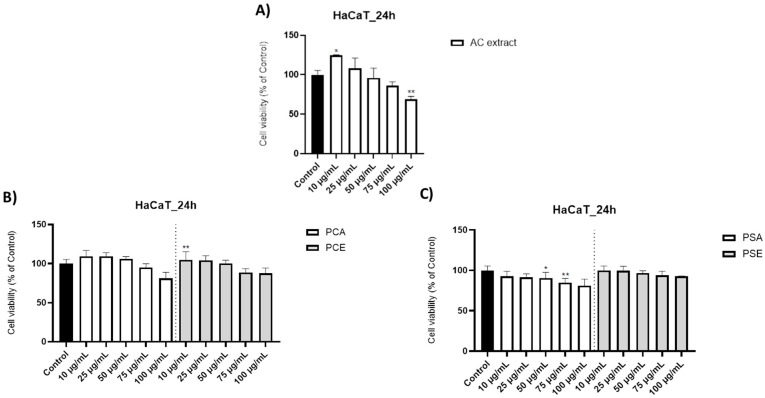
Graphical representations of the effect induced by (**A**) AC methanolic extract, (**B**) PCA and PCE, (**C**) PSA and PSE (at 10–100 µg/mL) treatment on HaCaT cells viability after 24 h of exposure. The results are expressed as percentages normalized to control (untreated cells) and are presented as mean values ± standard deviation (the experiments were performed in triplicate). To evaluate the statistical differences between the control and treatment groups, a one-way ANOVA test was conducted, followed by Dunnett’s multiple comparison post-test. “*” indicates statistical significance (* *p* < 0.05, ** *p* < 0.01). The control group results are depicted in black. The result after 24 h of exposure are depicted in grey.

**Figure 2:**
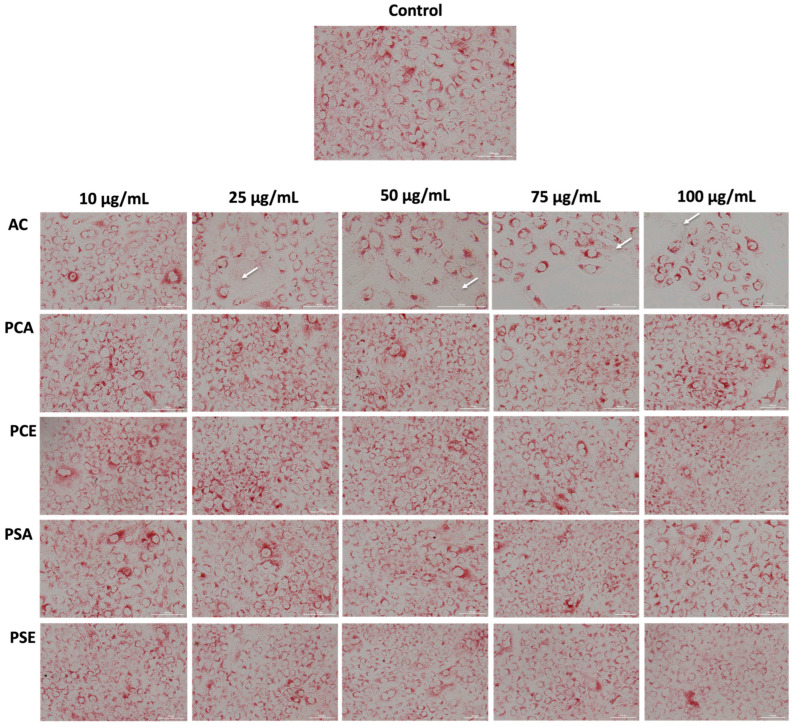
Neutral Red staining of HaCaT cells treated 24 h after the treatment with AC, PCA, PCE, PSA, and PSE 10–100 μg/mL. The white arrows indicate a decrease in NRU. The scale bar indicates 100 µm.

**Figure 3:**
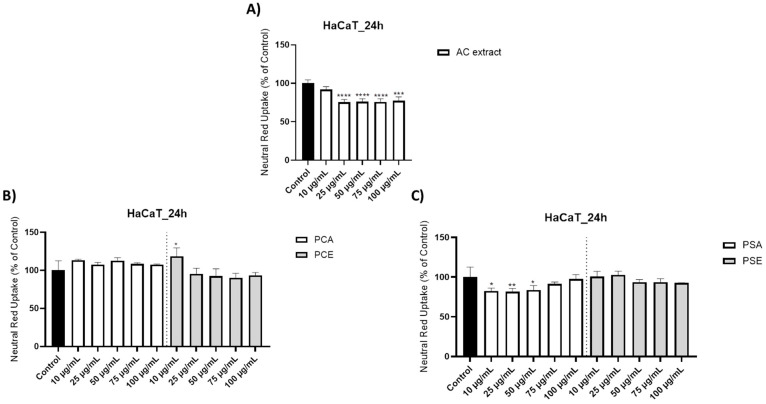
Graphical representations of neutral red uptake percentages in HaCat cells post-exposure to AC methanolic extract (**A**), PCA and PCE (**B**), and PSA and PSE (**C**) at 10–100 µg/mL. The results are expressed as percentages normalized to control (untreated cells) and are presented as mean values ± standard deviation (the experiments were performed in triplicate). To evaluate the statistical differences between the control and treatment groups, a one-way ANOVA test was conducted, followed by Dunnett’s multiple comparison post-test. “*” indicates statistical significance (* *p* < 0.05, ** *p* < 0.01, *** *p* < 0.001, **** *p* < 0.0001). The control group results are depicted in black. The result after 24 h of exposure are depicted in grey.

**Figure 4:**
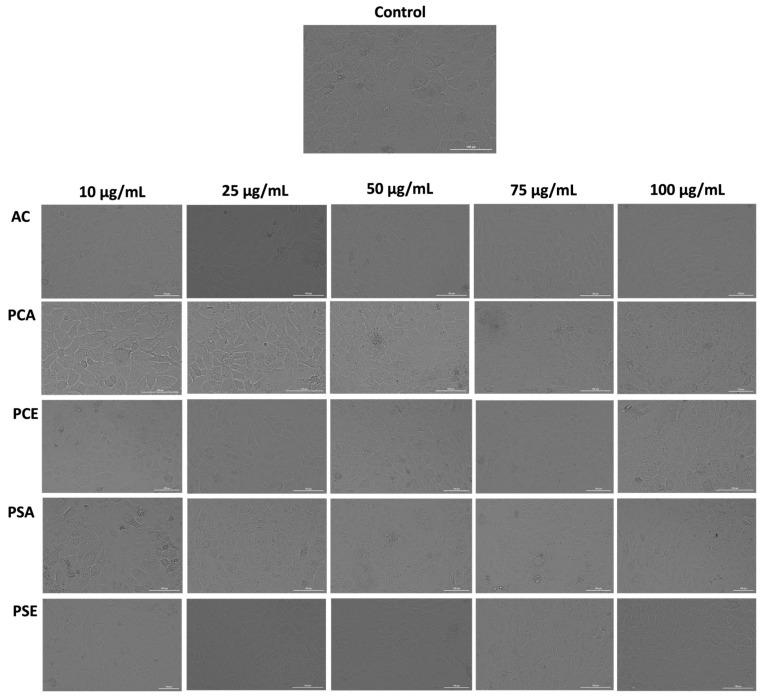
Representatives bright-field images with HaCaT cells 24 h after the treatment with AC, PCA, PCE, PSA, and PSE 10–100 μg/mL. The scale bar indicates 100 µm.

**Figure 5:**
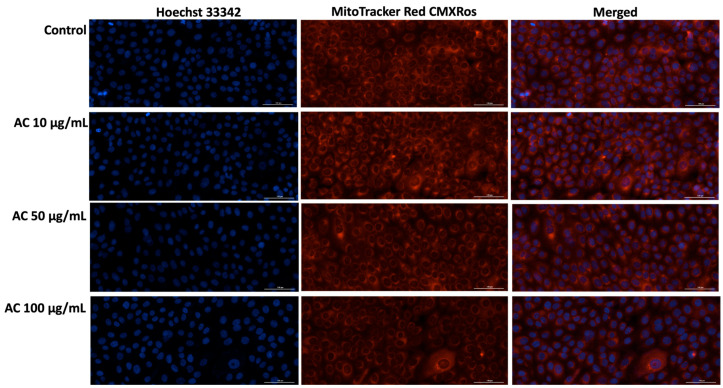
Nuclear and mitochondrial appearance of HaCaT cells treated for 24 h with AC 10, 50 and 100 μg/mL. The scale bar indicates 100 µm.

**Figure 6:**
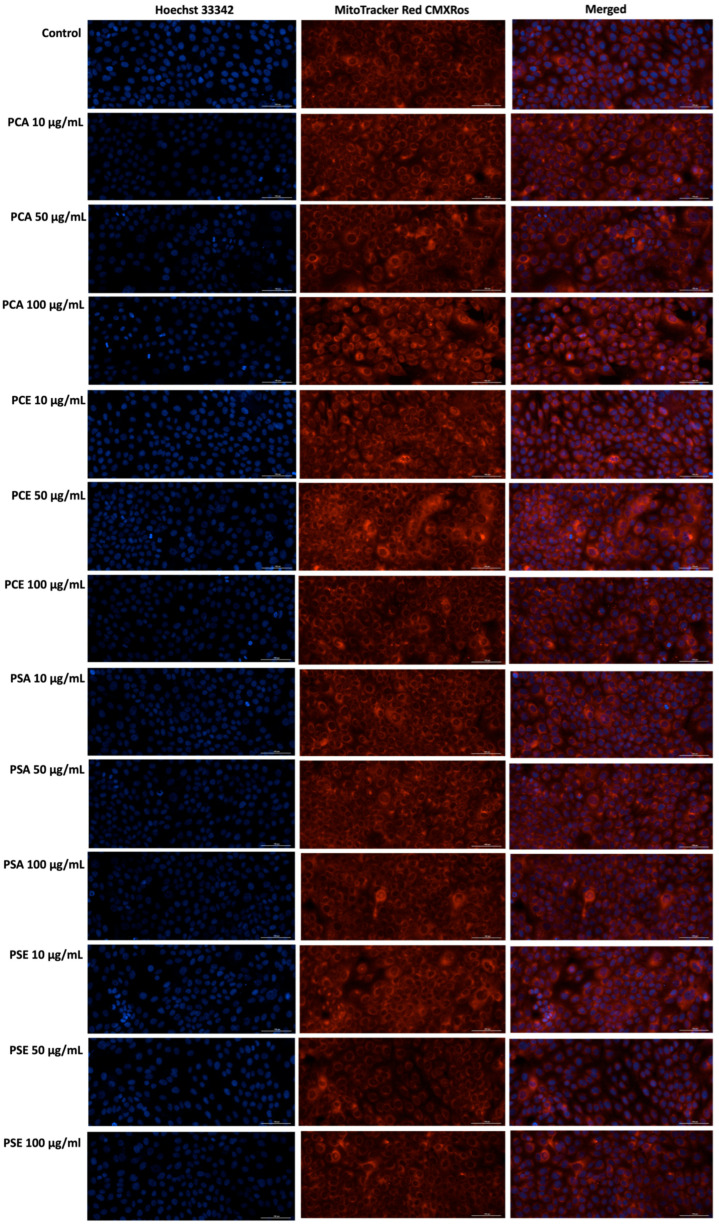
Nuclear and mitochondrial appearance of HaCaT cells treated for 24 h with PCA, PCE, PSA and PSE 10, 50 and 100 μg/mL. The scale bar indicates 100 µm.

Cell viability and Neutral Red uptake assays (Figure 1, Figure 2 and Figure 3) indicate that AC extract and the corresponding liposomal formulations do not induce pronounced cytotoxic effects at low and moderate concentrations (10–50 µg/mL), while exposure to higher concentrations causes dose-dependent but moderate reductions in cellular metabolic activity. Morphological evaluation by bright field microscopy (Figure 4), correlated with nuclear and mitochondrial analysis (Figure 5 and Figure 6), confirms the maintenance of the overall cellular architecture and the absence of major alterations of essential cellular compartments. These observations indicate a favorable biocompatibility profile, consistent with the predominance of isoquercitrin and the presence of phenolic acids in the AC extract.

This correlation may support the potential applicability of AC extract and its formulations in dermato-cosmetic and biomedical preparations, where studies regarding antioxidant activity, microbial control and compatibility with skin cells are required.

### 3.2. Determination of Total Flavonoid Content

The method used is based on the reaction of AlCl_3_ in an acidic medium with the ketone group in the structure of quercetin or with phenolic hydroxyl groups, forming colored complexes that can be quantified by spectrophotometry. After measuring the absorbance of the methanolic AC extract at 510 nm, the concentration of total flavonoids is calculated using the calibration curve equation and expressed as mg quercetin equivalents (QE)/g DW. The mean flavonoid concentration of the AC extract is 67.23 ± 0.33 mg QE/g DW.

The result obtained is consistent with findings reported by other researchers: 13.53 ± 0.15 mg QE/g ethanolic extract of dried leaves [8], 295.8 ± 12.6 mg QE/g ethanolic extract of root [59], 60.83 ± 0.01 mg QE/g DW leaves [7], 23.87 ± 3.33 mg QE/g dried plant [60].

### 3.3. Determination of Total Polyphenol Content

After measuring the absorbance of the methanolic AC extract at 765 nm, the total polyphenol concentration was calculated using the calibration curve equation and expressed as mg gallic acid equivalents (GAE)/100 g dry sample. The determinations were carried out in triplicate, and the results are expressed as mean value ± SD. The total polyphenol content measured comes out at 64.38 ± 0.16 mg GAE/g DW. Based on the data obtained, it can be concluded that the AC extract has a considerable total polyphenol content (64.38 ± 0.16 mg GAE/g dried leaf or 1.1287 mg GAE/mL methanolic extract).

The result is consistent with other determinations reported by researchers: 93.62 ± 1.63 mg GAE/g methanolic leaf extract [8], 91.3 ± 4.75 mg GAE/g ethanolic root extract [59], 67.26 ±0.24 mg GAE/g DW [61], 3.55 ± 0.06 mg GAE/g DW leafs [7], 93.62 mg GAE/g ethanolic extract of dried leaves [8], 1863 mg GAE/mL ethanol extract from the aerial part, undried product [62]. The differences in the amounts of total polyphenols in different types of extracts could be explained by the fact that the presence of phenols is affected by plant species, harvest maturity, growing conditions, soil conditions and post-harvest treatments [63].

### 3.4. Evaluation of the Antioxidant Capacity of Hydroalcoholic Extract of AC

Oxidative stress is a key factor contributing to epidermal damage induced by ultraviolet radiation, environmental pollutants, and microbial aggression, leading to inflammation, skin barrier damage, and premature aging. Reactive oxygen species (ROS) generated under these conditions overwhelm endogenous antioxidant defenses, especially in the superficial layers of the skin [64,65]. In this context, radical scavenging activity measured by DPPH and ABTS assays, which are based on chemical reactions, is accepted as a preliminary indicator of the potential of bioactive compounds to neutralize ROS relevant to cutaneous oxidative stress [66,67].

The observed DPPH and ABTS scavenging capacities suggest that the extract contains electron or hydrogen-donating compounds, such as polyphenols and flavonoids, which are known to contribute to epidermal protection by reducing oxidative damage and modulating redox-sensitive inflammatory pathways [24,68]. From the perspective of topical formulations, antioxidant activity is particularly relevant, since compounds applied to the skin surface act mainly in the stratum corneum and viable epidermis, where oxidative stress is most pronounced [69]. Furthermore, liposomal encapsulation has been reported to improve the stability and local availability of antioxidant compounds in topical delivery systems, potentially enhancing their protective effects against epidermal oxidative stress [70,71].

The flavonoids present in the extract of the AC plant are compounds that have antioxidant capacity and can neutralize free radicals.

#### 3.4.1. DPPH Method (2,2-Diphenyl-1-picrylhydrazyl Method)

Inhibition of 50% of the DPPH radical (IC_50_) is considered the reference point, and a lower IC_50_ value reflects a higher antioxidant capacity. To determine the IC_50_, several concentrations of methanolic extracts (0.1–0.5 mg/mL) were prepared, and for each concentration, the percentage of DPPH inhibition was measured. The antioxidant capacity of the AC plant extract samples was expressed as the percentage of DPPH radical inhibition, and the result is presented as the arithmetic mean ± SD. Thus, the equation describing the variation in the inhibition percentage for different concentrations of AC extract solution is:

y = 144.470x + 26.615, R^2^ = 0.9954 (6)

where y is the percentage of DPPH radical inhibition and x is the concentration of the methanolic AC extract samples. This equation was used to determine the IC_50_. Based on triplicate determinations, an IC_50_ value of 0.1619 mg/mL extract was obtained, corresponding to an inhibition percentage of 161.867 μg/mL extract.

Lower IC_50_ values, therefore a high antioxidant activity, was found for the methanol plant extract, 141.04 ± 0.25 μg/mL [61], 50.66 ± 0.14 μg/mL raw plant extract [8], 125.40 ± 2.40 μg/mL root extract of *Aristolochia bodamae* [72]. A determination of DPPH was reported as 3106 mM equivalent Trolox/L ethanolic extract from the aerial part, undetected [62].

From the comparison of these results we can conclude that we recommend that extractions be carried out from different parts of the plant product of AC, dried and unsalted.

#### 3.4.2. ABTS Method

The calibration curve was prepared using Trolox standard solutions of different concentrations. First, the blank (ABTS^+^ only) was measured. The Equation (7) obtained from the calibration curve describing the variation in ABTS^+^ inhibition percentage as a function of Trolox concentration was:

y = 84.01x + 4.42, R^2^ = 0.9989 (7)

where y is the inhibition percentage (%) and x is the concentration of the extract expressed as mg Trolox equivalent (mg TE/mL). Thus, the extract concentration was 0.152 ± 0.40 mg TE/mL extract.

To determine the IC_50_ inhibition percentage of ABTS^+^, the inhibition percentages of the ABTS radical were measured for extract samples of different concentrations of the methanolic AC extract solution, the calibration line being in Equation (8):

y = 161.16x + 16.87, R^2^ = 0.9954 (8)

where x—the concentration of the methanolic extract of AC, and y—the percentage of inhibition of ABTS^+^. Thus, the IC_50_ ABTS is 0.20557 mg/mL extract or 205.57 μg/mL extract.

Other similar results have been reported by other studies: 160.89 ± 0.21 μg/mL extract [7], IC_50_ 65.23 ± 2.49 *Aristolochia longa* from Aristolochia longa root extract [73].

From the comparison of these results we can conclude that perhaps extractions should be made from different parts of the plant product of AC, dry and unsalted.

The antioxidant capacity of the plant extract was evaluated in vitro by the chemical and cellular DPPH and ABTS methods. According to the literature, these methods mainly evaluate the free radical scavenging capacity and do not directly reflect the biological antioxidant activity in cellular or tissue systems [74,75].

The IC_50_ values determined for AC extract by both the DPPH and ABTS methods indicate a relevant capacity to neutralize free radicals in an acellular environment, but their biological significance must be interpreted in correlation with cellular signaling mechanisms and the response to oxidative stress. Rana J.N. et al., show that flavonoids and polyphenols do not manifest their antioxidant effect directly, but through complex biological effects that include the modulation of signaling pathways involved in redox homeostasis, inflammation, cell survival and apoptosis [76].

Isoquercitrin, identified as the dominant flavonoid in the extract, is a quercetin glycoside whose biological effects are distinct from the aglycone and other flavonols. Glycosylation at the 3-O position modulates solubility, membrane permeability, and cellular uptake, which in turn influence both antioxidant and antimicrobial activity [21,77]. Isoquercitrin and phenolic acids identified in AC extract can influence the activity of transcription factors sensitive to oxidative stress (e.g., Nrf2—in neuroinflammation or oxidative stress) [78,79]. Cerebral ischemia or reperfusion [80] can regulate the expression of endogenous antioxidant enzymes (superoxide dismutase, catalase and glutathione peroxidase) and can modulate the activation of MAPK pathways [81] as demonstrated for similar flavonoids (prunin) in the literature. Mechanistic studies indicate that isoquercitrin can scavenge reactive oxygen species, chelate transition metals, and modulate redox-sensitive signaling pathways in a manner that may differ from methylated or unglycosylated flavonols [77,82]. Similarly, its antimicrobial effects may be enhanced or modulated by the delivery format, as liposomal encapsulation may enhance local concentration and interaction with microbial membranes [82].

Therefore, the acellular antioxidant activity assessed by DPPH and ABTS methods in the present study should be considered a screening step, which can constitute the basis for further investigations oriented towards cellular models, relevant for elucidating the biological mechanisms of AC extracts. The integration of these data into a broader biological framework, as recommended by recent literature, emphasizes the need for functional validation through cellular and biological studies, in order to establish their translational relevance and the real therapeutic potential of AC extract. Overall, the study indicates an antioxidant potential supported by a favorable cellular tolerance profile, but further investigations are needed to elucidate the molecular mechanisms involved.

### 3.5. Liposome Preparation

Liposomes, due to their amphiphilic structure formed by loading polyphenols into the phospholipid membrane, cross various hydrophilic environments (including the gastrointestinal one), maintaining the stability of the bioactive compounds. Thus, the hydrophilic component of the liposomes interacts with the aqueous environment, while the lipophilic component facilitates integration through the cell membrane. This amphiphilic structure favors transmembrane diffusion and increased cellular absorption of polyphenols. Subsequently, liposomes allow the efficient release of bioactive compounds into the general circulation, leading to a superior bioavailability compared to the forms not bound to phospholipids [83,84]. The natural liposomal formulations exhibit substantial scientific advantages over basic synthetic surfactants in biomedical drug delivery contexts. These include superior biocompatibility, biodegradability, functional delivery performance, targeted release, and reduced systemic side effects—attributes underpinned by their biomimetic structure and metabolic compatibility. Meanwhile, synthetic surfactants remain valuable in less demanding roles (e.g., formulation solubilization), but lack the multifunctional capabilities required for advanced therapeutic delivery systems [85].

To protect it from external factors that could oxidize it, dry AC extract was introduced into two types of liposomes. These phospholipids were chosen because they are recommended for human use, being composed of chains of superior fatty acids with different lengths and degrees of saturation, representing the lipid membrane units of liposomes [86]. Beyond their role as structural excipients, phospholipid composition critically determines the biological behavior of liposomal carriers [14]. Phosphatidylcholine (PC), a zwitterionic phospholipid and a major constituent of mammalian cell membranes, is known to confer high membrane fluidity, biocompatibility, and stability to lipid vesicles, favoring passive fusion and sustained release profiles [15,87]. In contrast, phosphatidylserine (PS), an anionic phospholipid physiologically localized on the inner leaflet of cell membranes, introduces a negative surface charge that can significantly influence vesicle–cell interactions.

PS-containing liposomes have been reported to exhibit enhanced interactions with keratinocyte membranes through electrostatic attraction and recognition by PS-binding proteins, potentially promoting increased cellular uptake and intracellular delivery [88,89]. Moreover, variations in phospholipid composition modulate bilayer packing, membrane rigidity, and permeability, thereby affecting release kinetics and the intracellular bioavailability of encapsulated bioactive compounds. Consequently, the selection of PC versus PS as membrane-forming lipids is expected to impact not only physicochemical stability but also keratinocyte-specific biological responses, including cytoprotection and modulation of oxidative and inflammatory pathways [15,24].

Taking into account the solubility of phospholipids, we have chosen the right organic solvents. Thus, colloidal particles were obtained with a membrane composed of phospholipids and cholesterol, loaded or not with AC extract.

To hydrate the lipid film, a phosphate buffer with a pH of 7.4 was used, a buffer that offers advantages: spontaneous formation of liposomes at neutral pH, negative charge of liposome membranes, which gives them greater stability. The addition of cholesterol involves modeling the stiffness of lipid membranes and the stability of liposomes [90].

Studies in the literature show that after hydration of the lipid film, giant multilamellar liposomes are obtained, and through sonication and centrifugation, they shrink in size [91]. Thus, four liposomal formulas were obtained, two loaded with AC extract, two unloaded.

### 3.6. The Entrapment Efficiency

The entrapment efficiency was determined using UV-VIS spectrophotometry, employing the same calibration curve used for determining the total flavonoid content in the dried AC extract. The AC extract samples were measured in triplicate; therefore, the results are expressed as mean value ± SD (standard deviation). The data obtained for the entrapment efficiency are presented in Table 3.

Entrapment efficiency (EE) quantifies the fraction of polyphenols that is incorporated into liposomal vesicles relative to the total amount added during formulation. EE values above 80% are commonly reported for liposomal bioactive compound formulations (luteolin or isoscutellare in liposomes with EE up to ~95%) because these compounds are lipophilic and distribute favorably in the lipid bilayer, rather than remaining in the aqueous phase [92].

From the analysis of the data in Table 3, a high entrapment of the extract in the liposomes was observed, with minimal variation between the two liposomal formulations, even though different phospholipids were used. Thus, for both liposomal formulas, the entrapment efficiency values ranged between 82.68 and 84.33%, values that did not significantly decrease over the 30-day period, indicating that the synthesized liposomes are stable. The highest entrapment efficiency was obtained for the liposome containing phosphatidylcholine.

This finding is consistent with other studies [93], which showed that when liposomes are formed, lipid composition does not greatly influence entrapment. Some researchers introduced *Stellaria media* (L.) Vill. extract into liposomes, and entrapment efficiency was 92.09% and 84.25%, using the same phospholipids, phosphatidylcholine and phosphatidylserine [94].

### 3.7. In Vitro Release Studies of the AC Extract

The in vitro release of the AC extract was evaluated by determining the amount of total flavonoids released over time, and the results are presented in Table 4. The same calibration curve used for determining the entrapment efficiency was applied here as well.

For successful topical delivery, a formulation must overcome the skin’s diffusion barrier, chiefly the stratum corneum. Liposomal carriers facilitate: occlusion and hydration of skin, increasing drug partitioning into the epidermis; interaction with intercellular lipids of the skin, which can enhance diffusivity [95]. A biphasic release profile typically consists of a relatively rapid release in the first few hours, followed by a slower, sustained release that extends over 24 h or more. Thus, early release (diffusion-dominated) is driven primarily by the diffusion of biocompound molecules that are near or on the surface of the liposomal membrane or are loosely associated with the outer bilayer. After this initial stage, release becomes slower as the lipid membrane of the vesicle progressively reorganizes or relaxes. A relaxation or restructuring of the phospholipid membrane occurs, which controls the rate at which encapsulated biocompounds are evacuated from the vesicle core [96,97].

From the analysis of the data in the table it can be seen that the release of trapped flavonoids occurs gradually, but the largest amount occurs in the first 8 h, after which the yield increases very little, the curve becoming almost parallel to the axis of the abscissa.

The percentage of flavonoid release from liposomes in vitro, in Franz diffusion cells, after 48 h is very close for the two types of liposomes, but the largest amount is released by the PSA liposome. The largest amount of trapped flavonoids was released in the first 5 h (over 50%), the next three hours the yield occurred at a slower rate, and after that the percentage of release remained almost constant.

With the passage of time, the percentage of yield of flavonoids trapped in the two liposome formulas increases. Following the results obtained, the highest percentage of flavonoid yield after 48 h was the PSA liposome.

#### 3.7.1. AFM Analysis of Liposomes

AFM images of the PSA, PCA, PSE and PCE sample, together with extracted roughness data were obtained. Calculated values from AFM images (Average roughness (Sa), Mean Square Root Roughness (Sq), Maximum peak height (Sp), Maximum valley depth (Sv), Maximum peak-to valley height (Sy), Surface kurtosis (Sku), Surface skewness (Ssk)) are shown in Table 5. To assess the stability of the four types of synthesized liposomes, measurements were performed on day 1 (immediately after preparation), day 15, and day 30.

At the analysis by AFM, for liposomes loaded with AC extract and naked liposomes, it is observed that the results obtained show similar values, slightly increased for Sa, Sq, Sp, Sv, Sy, in the determinations that were made at 15 and 30 days, respectively, compared to the first determination. These changes are very small. Taking into account that the force with which the tip is pressed on the surface and the distance between the tip and the surface have the same values, these small increases may be due to the nature of the analyzed sample and the wear of the tip of the apparatus used [98].

Four samples consisting of two base materials (PSE and PCE) and two with additional medicinal substance (PSA and PCA) were analyzed. The obtained results revealed the highest roughness values of 71.00 nm (Sq, respectively, 58.22 nm (Sa) in case of PCA among all compared samples. However, in case of base materials, sample PCE stands out with 24.17 nm (Sq), respectively, 19.42 nm (Sa). When comparing the appearance of the samples, more homogenous surface is observed when added medicinal substance, covering the uneven surface of the base material (Figure S1). However, the roughness is higher given the increase in layer thickness and the increase in Maximum peak height (Sp) value and Maximum valley depth (Sv) value. This occurrence is probably also due to the occasional disruptions between the new added layer and old layer, creating impression of deeper valleys, which may also be present due to the deposition method (drop casting). The Maximum peak-to valley height (Sy) value was obtained from the sum of Sp and Sv. Therefore, bigger Sp, respectively, Sv value, bigger the Sy value. The Sy value is also referring to the Z value from the 3D images, indicated in Figure S1 (Supplementary Material). Sku and Ssk are additional parameters involving the aspect of the surface, whereas Sku describes if the majority of the heights, respectively, valleys, are steep (Sku > 3) or rounded (Sku < 3), while Ssk highlights the dominant distribution of valleys (negative Ssk, respectively, heights (positive Ssk) along the surface [99]. In case of all four samples, the Sku is below 3, suggesting the prevalence of neither steep or rounded profile surface. Regarding the Ssk values, all samples indicate positive Ssk, however since the values are around 0, it suggests a harmonious distribution between valleys and heights. The images depicting profile (Figures S2–S5, (Supplementary Material)) on the specific areas is in accordance with the Sku and Ssk values. In the Figures S2 and S3, the highest heights of the registered profile area are around 120–140 nm, whereas the surrounding profile area is between 40 and 60 nm which corresponds to the heights registered on the base materials in the Figures S4 and S5.

#### 3.7.2. DLS Analysis

Studies on liposomes have shown that their size is a parameter influencing pharmacokinetics, tissue distribution, hepatic absorption and accumulation, diffusion through tissues, and renal excretion.

To characterize the liposomes loaded with AC extract, a macroscopic analysis of the suspensions containing the synthesized liposomes was first performed. The suspension containing PCA liposomes has been observed to have a milky, yellow-orange appearance, and the suspension containing PSA liposomes has a milky, pale yellow appearance. Suspensions containing unfilled liposomes have a milky appearance, but are lighter in color.

The diameter of the liposome is an important parameter that influences the behavior of liposomes both in vitro and in vivo and their stability over time [100]. Thus, the specialized literature has shown that when liposomes are “giant,” they exhibit better in vivo performance compared to liposomes with nanometric size [101]. This can be explained by the fact that “giant” liposomes persist in the blood longer compared to those that are nanometric in size, which recommends that they be administered at a longer interval of time [94].

To determine whether the incorporation of the AC extract altered the size of the liposomes, we compared the percentage distribution of liposomes within specific size intervals for the two types of loaded liposomes versus their corresponding empty liposomes. The liposome histograms show that the size distribution of both loaded and empty liposomes is present in approximately the same proportion within the 100–1000 nm range. This demonstrates that the inclusion of the AC extract does not modify the liposome diameter. Thus, PSA liposomes exhibit a diameter below 1000 nm similar to PSE liposomes (66.616% versus 67.920%), and PCA liposomes present a similar proportion of vesicles below 1000 nm as PCE liposomes (80.177% versus 76.109%). Considering the relatively high percentages, it can be concluded that both types of liposomes—empty and loaded with AC extract—belong to the category of “giant” liposomes. Comparative diameter distribution histograms for loaded and unloaded liposomes are shown in Figures S6 and S7 (Supplementary Material).

Studies on liposomes have shown that when the molecular weight of the phospholipid used in their preparation is high, it influences their size, surface charge, and shape. Thus, the smaller diameter of PS and PSE liposomes can be explained by the fact that phosphatidylserine has a lower molecular weight compared to phosphatidylcholine, which has a bulkier molecular structure. Typically, the net charge of the particles corresponds to the surface charge, or Zeta Potential [102]. This parameter of liposomes is important to establish the electrostatic interactions between the particles in the suspension [103]. The charge of liposomes is determined by the type and lipid composition used in their preparation, and it can be negative, positive, or neutral. It has been observed that when the charge has a low absolute value (below −30 mV or above +30 mV), the liposomes tend to aggregate over time because there are insufficient repulsive forces between them, which leads to their flocculation [104]. It can be observed that the molecular structure of the phospholipid also influenced the electrical charge of the liposomes. Negative Zeta Potential values were obtained for all liposomes, both the loaded and the empty ones.

To determine the stability of the liposomes over time, they were stored for 30 days at room temperature, in dark-colored bottles, and analyzed again, maintaining the same analysis conditions. The data obtained are shown in Table 6, as average values ± DS.

Natural lipids contain a hydrophilic part, the head, and one or more hydrocarbon chains that are hydrophobic. Overall, lipids are electrically neutral, with a zeta potential between −10 and +10 mV [105], as has been achieved for empty liposomes, regardless of the type of lipid used. Analyzing the values obtained for the Zeta Potential for the two types of charged liposomes and the two types of uncharged liposomes, it is observed that when loaded with AC extract, the Zeta Potential decreases, becoming anion liposomes. However, because the zeta potential value is not more negative than −30 mV or more positive than +30 mV, the suspension is not stable because there is not enough electrostatic repulsion between its molecules, which would lead to a higher flocculation tendency [106]. Zeta potential values between −5 mV and −18 mV indicate poor to moderate colloidal stability and have important implications in pharmaceutical, cosmetic, or nanotechnological formulations, where particle dispersion plays a critical role.

Thus, values between −5 and −18 mV indicate a system with a tendency to aggregate over time, in the absence of stabilizers (surfactants, polymers, etc.) or possibly unstable in storage or under variable conditions of pH, temperature, shear, or electrostatically unstable, possibly requiring additional steric stabilization.

In the case of liposomes or particles for topical administration, values around –10 mV may be acceptable if there is steric stabilization (e.g., with PEG, polysaccharides). They can be tolerated if the product life is short or if interaction with the target tissue is prioritized over long-term stability.

However, when comparing the Zeta Potential values for the two types of charged liposomes, it is observed that PSA liposomes are more stable than PCA liposomes. Even if, according to the Zeta Potential which is not very high, the tendency to flocculation is relatively high, however, after 30 days, it does not show great changes, which means that the liposomes have not clumped in suspension.

The PDI value can vary between 0.0 (perfectly uniform sample in particle size) and 1.0 (very polydisperse sample with multiple particle sizes). If the PDI value for liposomes is between 0 and 0.5, this indicates a homogeneous distribution of the size of the liposomes [107,108]. According to the data presented in Table 4, the PDI for liposomes synthesized at baseline (T0) ranged from 0.216 ± 0.10 to 0.348 ± 0.32, and after 30 days of storage, the values ranged from 0.278 ± 0.16 to 0.483 ± 0.29. This variation indicates a relatively uniform distribution of liposome size, with a slight increase in heterogeneity over time, possibly due to aggregation processes or colloidal instability.

Comparatively, the empty liposomes showed PDI values lower than or equal to 0.3, which suggests a uniform size distribution characteristic of monodisperse systems. When loaded with AC extract, the liposomes exhibited a PDI of approximately 0.5, indicating a relatively uniform size distribution, with a slight increase in heterogeneity over time, possibly due to aggregation processes or colloidal instability. This phenomenon may be attributed to interactions between the extract components and the liposomal membrane, which can affect the physical stability and homogeneity of the formulation by altering the structure or surface charge of the liposomes [107]. In general, a polydispersity index below 0.3 is considered optimal for liposomes; however, PDI values < 0.5 are still acceptable for complex formulations, such as those loaded with active substances, and are regarded as suitable for pharmaceutical or cosmetic applications as long as the overall system stability is maintained. Tu Q. et al. reported that the PDI values for cinnamon-loaded liposomes obtained from chitosan and sodium alginate via the electrostatic layer-by-layer self-assembly technique were below 0.4, indicating a uniform size distribution of the liposomes [109]. Gu Y. et al. reported that the PDI of liposomes loaded with bamboo leaf flavonoids and coated with alginate and chitosan was 0.36, suggesting that the liposomes exhibited a homogeneous dispersion [110].

The measured ZP values, together with the PDI values, indicate that the liposomal formulations have limited electrostatic stabilization and moderate polydispersity even at the time of preparation. Such a profile suggests a relatively high tendency towards particle aggregation, consistent with the observed increase in PDI over time, reflecting progressive colloidal instability. Although these formulations may be practically applicable for short-term or immediate topical use, their physicochemical characteristics highlight the need for further optimization—such as surface modification, incorporation of steric stabilizers or adjustment of lipid composition—to enhance long-term stability and reduce aggregation [18,19,111,112,113].

For topical applications, short-term stability may still be acceptable as products are often used promptly and stored under controlled conditions. However, the observed values highlight the importance of monitoring formulation stability over time to ensure consistent performance [18,19,111].

### 3.8. Microbiological Analysis

It was determined that AC leaf extract had an effect on the reference strains: *S. aureus*, *E. coli*, *P. aeruginosa*, *C. albicans*. The results obtained are shown in Table 7 and Figure S8 (Supplementary Material).

*S. pneumoniae* is a Gram-positive bacterium whose cell wall integrity is essential for its survival and pathogenicity. *S. aureus* is also a Gram-positive bacterium, well known for its antibiotic resistance, especially in MRSA (Methicillin-resistant *Staphylococcus aureus*) strains. *E. coli* is a Gram-negative bacterium frequently associated with intestinal and extraintestinal infections; it possesses a complex cell membrane structure, including an additional outer membrane that can influence the mechanism of action of polyphenols and flavonoids. *P. aeruginosa* is a pathogenic Gram-negative bacterium resistant to numerous antibiotics, with the ability to form biofilms [114].

After analyzing the results in Table 7, it can be concluded that the AC extract and the liposomal solutions (PCA, PSA) exhibit antimicrobial activity against some of the tested standard strains (*S. aureus*, *S. pneumoniae*, *E. coli*, *P. aeruginosa*) as well as certain wild strains, but they do not act against *Candida albicans* ATCC 90029.

There are some studies that have attributed antimicrobial activity to very high concentrations of aristololic acid I and aristololic acid II [115], and other studies that concluded that although the solvent used in the extraction gave the extract with the highest concentration of aristololic acid I and aristololic acid II, yet this extract did not have antimicrobial effects on the strains investigated [116]. Probably, this effect could be due to the action of polyphenols and flavonoids contained in the extract of the plant product.

In other studies, it has been reported that the methanolic extract of AC showed activity against *E. coli*, similar to extracts obtained with other solvents from *A. bracteolata* [117]. Methanol, hexane, and ethyl acetate extracts from *A. clematitis* leaf showed similar efficacy against both *S. aureus* strains, but at a higher concentration (2 mg/mL) compared to the leaf extract of *A. bracteolata* [118]. From studies conducted on extracts obtained from different parts of the plant material of various *Aristolochia* species, it has been concluded that they exhibit antimicrobial effects against *S. aureus* strains. The most effective antimicrobial activity against *S. aureus* strains was detected in fruit extracts. This bacterium frequently causes skin and wound infections. In the case of the AC extract, this effect may be attributed to the presence of polyphenols, saponins, and tannins. In general, these compounds can be found, in varying concentrations, in extracts obtained using different organic solvents, whether polar or non-polar, resulting in extracts with differing antimicrobial activities [59].

There are studies that have reported that the action of polyphenols and flavonoids on various strains of bacteria is achieved through several mechanisms. Thus, by inhibiting the processes involved in the formation of the cell wall of bacteria, it makes them more exposed to external attacks and even cell death in *S. pneumoniae* [119,120,121], in *S. aureus* [114,119,121,122], in *E. coli* [119,122].

Another mode of action is to affect the integrity of the cell membrane, by modifying/increasing their permeability, which would lead to the modification of the cell content, preventing growth and even the destruction of bacteria *S. pneumonie* [121], *S. aureus* [114,122], *E. coli* [123,124,125], *P. aeruginosa* [119,122].

Some studies have shown that another mechanism of action is to inhibit the activity of bacterial enzymes essential for metabolization and virulence processes (proteases and lipases), which give bacteria the ability to invade and infect host tissues in *S. aureus* [123], in *P. aeruginosa* [119,120,122].

Polyphenols and flavonoids can alter the expression of bacterial virulence genes, thereby reducing the ability to produce toxins or adhere to host surfaces for *S. pneumoniae* [119,122,126], *S. aureus* [119,122,126], *E. coli* [127], *P. aeruginosa* [120,128,129]. Polyphenols and flavonoids can also inhibit the formation of biofilms, a protective structure that helps bacteria resist antibiotic treatments and immune system attack, making it easier to eradicate themselves from the body. This mechanism has been reported for bacteria as: *S. pneumonie* [121], *E. coli* [122,126,130], *P. aeruginosa* [122,127].

### 3.9. In Vitro Evaluations

Consequently, this work was further extended with in vitro evaluations aimed at investigating the cytocompatibility and cellular tolerability of the samples in skin-derived cells. In this context, to assess the topical biosafety and cytotoxic potential of all the samples of interest, the HaCaT 2D cell line was employed for the in vitro evaluations. The assays were performed after a 24 h incubation period following treatment, an exposure interval commonly applied in HaCaT-based studies investigating plant-derived extracts and cutaneous safety [131,132,133,134]. Furthermore, these cells exhibit morphological and functional characteristics comparable to those of isolated keratinocytes. These cells represent the primary functional and structural components of the epidermis and are specialized in protecting the skin against external stressors. Structural or functional abnormalities in keratinocytes constitute pathophysiological signs of skin diseases, including atopic dermatitis [135].

Cell Viability—For the assessment of the cytotoxic potential of the AC extract and its liposomal formulations at concentrations of 10, 25, 50, 75, and 100 µg/mL, a cell viability assay was conducted using the MTT method. Regarding the methanolic plant extract (AC), the cell viability data showed that at the lowest tested concentration (10 µg/mL), a slight stimulatory effect was observed, with viability being 124.54% relative to the untreated control cells. A similar increase was recorded at 25 µg/mL, where cell viability reached 107.89%. Starting from 50 µg/mL, a dose-dependent decrease in viability was recorded, with values of 95.99% at 50 µg/mL, 85.98% at 75 µg/mL, and 68.90% at 100 µg/mL (Figure 1A).

For the AC extract incorporated into phosphatidylcholine liposomes (PCA), the following cell viability values were obtained: 109.42% at 10 µg/mL, 109.16% at 25 µg/mL, 106.19% at 50 µg/mL, 95.07% at 75 µg/mL, and 81.06% at 100 µg/mL (Figure 1B). To exclude any viability effects attributable to the liposomal formulation, HaCaT cells were also treated with empty phosphatidylcholine liposomes (PCE). The corresponding viabilities were 104.90% at 10 µg/mL, 104.72% at 25 µg/mL, 100.39% at 50 µg/mL, 88.53% at 75 µg/mL, and 87.51% at 100 µg/mL (Figure 1B).

Concerning the AC extract incorporated into phosphatidylserine liposomes (PSA), the following viability values were recorded: 92.55% at 10 µg/mL, 91.56% at 25 µg/mL, 90.47% at 50 µg/mL, 84.96% at 75 µg/mL, and 80.88% at 100 µg/mL (Figure 1C). Treatment with the corresponding empty phosphatidylserine liposomes (PSE), yielded viability values of 99.89% at 10 µg/mL, 99.54% at 25 µg/mL, 96.63% at 50 µg/mL, 94.06% at 75 µg/mL, and 92.63% at 100 µg/mL (Figure 1C).

The MTT method is commonly used to determine the number of viable cells in proliferation and cytotoxicity studies. The technique is a low-cost approach based on the cleavage of yellow tetrazolium salt to form a soluble blue formazan product by mitochondrial enzymes, and the amount of formazan produced is directly proportional to the number of live cells present during exposure to MTT [136]. Notably, formazan formation is not only dependent on cell number, but also on the metabolic status and intracellular reducing capacity of viable cells. The reduction process involves NAD(P)H-dependent oxidoreductases and dehydrogenases, enzymes that are strongly influenced by mitochondrial functional state [137].

In this context, the present results on keratinocyte cells demonstrate that AC incorporated into both types of liposomes (PCA, PSA maintained cell viability within a range compatible with good cellular tolerance (all percentages of liposomes with AC reducing viability to only 80%). Empty liposomes also exhibited good biocompatibility toward HaCaT cells. However, these results indicate that cell viability was preserved. In parallel, mitochondrial metabolic capacity remained intact, suggesting the maintenance of cellular bioenergetic function. This aspect is particularly relevant given the essential role of mitochondrial metabolism in keratinocyte differentiation, epidermal homeostasis, cellular stress responses, and the formation and maintenance of the epidermal barrier [138,139].

Comparable approaches have been reported in the literature. Semenescu et al. also used the same method for determining cell viability, i.e., MTT, including on HaCaT cells, to identify the impact of certain natural extracts (*Galium verum* extracts) [140]. In another study conducted by Girija et al., an extract from another similar species, *Aristolochia bracteolata*, was evaluated on human dermal fibroblasts (HDF) and HaCaT cells, the latter also being used in our study. The results showed that *A. bracteolata* extract inhibited the proliferation of both cell types linearly and dose-dependently according to the cellular viability assay [131]. Since topical formulations are used repeatedly in practice, the absence of early cellular stress responses in vitro is relevant, as preserved cellular integrity is essential for sustaining the skin functions during continued exposure [141]. Furthermore, liposomes are already recognized in the drug industry and in the cosmetics industry for their ability to transport and protect encapsulated ingredients. They provide the benefit of improving the solubility of ingredients and controlling the release of agents. Several liposomes have already been used in the treatment of various conditions, including skin diseases such as melanoma [142].

Neutral Red Staining—Since the MTT assay revealed no significant cytotoxic effects after treatment with AC, PCA, PSA, or the empty liposomal formulations, and given that MTT mainly evaluates cell viability through mitochondrial metabolic activity [136]. The investigation was supplemented with the Neutral Red Uptake assay (NRU) to determine whether any cytotoxic responses might be associated with alterations in lysosomal integrity.

The microscopic evaluation following the NRU assay revealed that the tested formulations maintained lysosomal integrity, evidenced by the effective internalization of Neutral Red in a pattern comparable to the control, thus cells exposed to PCA, PCE, PSA, and PSE displayed a more intense intracellular staining and retained a cellular morphology closely similar to that of the untreated control. In contrast, cells treated with the methanolic AC extract showed a reduction in dye accumulation beginning at 25 µg/mL, accompanied by a decrease in cellular confluence (Figure 2).

The quantitative evaluation of NRU percentages for the AC extract (Figure 3A) showed the following values: 91.97% at 10 µg/mL, 75.57% at 25 µg/mL, 76.00% at 50 µg/mL, 75.74% at 75 µg/mL, and 77.31% at 100 µg/mL.

For the AC extract incorporated into phosphatidylcholine liposomes (PCA), the NRU assay yielded the following results: 113.27% at 10 µg/mL, 107.59% at 25 µg/mL, 112.86% at 50 µg/mL, 108.41% at 75 µg/mL, and 107.72% at 100 µg/mL (Figure 3B). The effect of empty phosphatidylcholine liposomes (PCE) was also assessed, with the following percentages recorded: 118.27% at 10 µg/mL, 95.40% at 25 µg/mL, 92.39% at 50 µg/mL, 90.20% at 75 µg/mL, and 93.51% at 100 µg/mL (Figure 3B).

Regarding the AC extract formulated in phosphatidylserine liposomes (PSA), the NRU values obtained at 10, 25, 50, 75, and 100 µg/mL were 82.55%, 81.56%, 83.81%, 91.63%, and 97.55%, respectively (Figure 3C). Treatment with empty phosphatidylserine liposomes (PSE) resulted in NRU percentages of 100.56% at 10 µg/mL, 102.87% at 25 µg/mL, 93.63% at 50 µg/mL, 93.56% at 75 µg/mL, and 92.63% at 100 µg/mL (Figure 3C).

Thus, the NRU evaluation indicates only a slight lysosome-associated cytotoxicity in HaCaT cells following treatment with the methanolic AC extract, with an NRU value of 77.31% at 100 µg/mL (Figure 3). In contrast, the liposomal formulations of the extract (PCA, PSA) and the empty liposomes (PCE, PSE) exhibited NRU values comparable to the control, suggesting an additional indication of their potential biocompatibility. This observation is further supported by the microscopic findings presented in Figure 3. At the level of keratinocytes, lysosomes are involved in critical cellular processes such as autophagy, maintenance of metabolic balance, support of mitochondrial function, and modulation of cellular responses to oxidative stress during the process of cellular differentiation. In addition to their involvement in these processes, lysosomes also contribute to the production of ceramides, key lipid components with protective roles in the epidermal barrier. Together, these functions highlight the importance of lysosomal integrity for skin homeostasis, while lysosomal dysfunction may lead to disturbances in epidermal barrier function [143].

The literature indicates that liposomal formulations are capable of being internalized at the cellular level via endocytic pathways and subsequently accumulating in lysosomal compartments. As a consequence, the use of the neutral red test, represents a relevant approach for evaluating the potential cytotoxic effects occurring at the lysosomal level following treatment with these formulations. Neutral red is selectively absorbed by lysosomes and other acidic organelles in cells [144,145]. The assay is based on the ability of viable cells to bind the cationic neutral red dye, which diffuses across the cell membrane and accumulates within lysosomes by interacting with anionic groups of the lysosomal matrix. This accumulation is dependent on the maintenance of the intracellular lysosomal pH gradient. When the lysosomal pH gradient is reduced comparativ to its normal physiological state, the capacity of the cells to retain the dye decreases accordingly reflecting a consequent reduction in cellular viability [41]. Following the same idea, Roggia et al. tested the stability and toxicity of guarana liposomes in different skin cell lines (HaCaT, Swiss 3T3 albino murine fibroblasts, and 1BR.3.G human fibroblasts). In addition to MTT, the research group also used neutral red, evaluating at intervals of 24, 48, and 72 h. The results indicated that guarana, empty liposomes, and guarana-containing liposomes produced only a slight reduction in cell viability (only at the highest concentrations), concluding that guarana-containing liposomes have the potential to be a promising system for topical applications [146].

Cell morphology—Figure 4 illustrates the morphology of HaCaT cells after 24 h of treatment with AC, PCA, PCE, PSA, and PSE in concentrations ranging from 10 to 100 μg/mL, compared to the control (untreated cells). The only change occurs with AC treatment, at the highest concentrations, where a slight decrease in confluence occurs compared to the control. According to the representative images, none of the samples analyzed induced alterations in cell morphology and structure compared to the control. The cell shape after treatments maintained its normal morphology, the same observed in untreated cells.

Cellular morphological analysis, including bright-field microscopy, is frequently used in research to visualize the impact of certain agents on cell lines. These experiments are key methods to reveal alterations in cell shape, thus playing a crucial role in the assessment of cytotoxicity [147]. The results of the morphological evaluation indicated the absence of cellular alterations such as cell rounding, detachment, or cellular shrinkage, together with the preservation of cell adhesion. Maintenance of cellular morphological integrity is important for preserving the structural integrity of the skin, while sustained cell–cell adhesion and normal cellular morphology support epidermal barrier function [148].

Mitochondrial and Nuclear Morphology Assessment—According to Figure 5, it can be observed that the nuclear and mitochondrial structure were not affected by 24 h of AC treatment. In addition compared to the control, a slight decrease in confluence was observed with increasing concentration.

Figure 6 shows that treatment with PCA and PCE did not induce nuclear and mitochondrial aberrations in keratinocytes cells compared to the control. The same trend can be observed in the case of PSA and PSE, which appeared safe and did not alter nuclear and mitochondrial structure.

The preserved mitochondrial structure and distribution observed by MitoTracker staining indicate the absence of early mitochondrial stress or structural damage following the treatment. This observation has an important relevance for topical formulations, as the skin is the largest organ of the human body, and mitochondria play a vital role in maintaining epidermal cellular functionality. Mitochondrial dysfunction has been associated with skin aging, increased susceptibility to cumulative cellular stress, whereas targeting mitochondria related processes has been shown to support skin rejuvenation and epidermal adaptive capacity [149]. The nucleus is a key indicator of cell death. Activation of apoptosis pathways, a form of which is programmed cell death, can lead to nuclear alterations and dysmorphologies. Typical apoptotic modifications include chromatin condensation, nuclear contraction, and the formation of apoptotic bodies [150]. In the present study, none of these characteristic apoptotic features were observed following treatment with any of the tested samples.

In relation to the antibacterial findings, the in vitro HaCaT results indicate a differential biological response. However, it is commonly reported in the scientific literature that plant-derived extracts can exhibit significant antibacterial activity while not inducing cytotoxic effects or structural alterations in HaCaT keratinocytes [151,152]. This selective behavior can be attributed to several factors. First, bacteria possess specific biological targets—such as the bacterial cell wall, bacterial membrane, biofilm and efflux pumps—that can be affected by phytocompounds, particularly polyphenolic and flavonoid compounds [153]. These structures are absent in eukaryotic cells, including HaCaT keratinocytes, which may explains the differential biological response. Moreover, the HaCaT cell line has been widely reported as relatively resistant to treatment with various natural compounds. This resistance has been attributed, among other factors, to the specific lipid composition of their membranes, which can contribute to maintaining cellular structure and functional integrity [152]. Furthermore, encapsulation into liposomal formulations has been shown to enhance the antibacterial activity of incorporated compounds due to the efficient interaction between the phospholipid bilayer structure and the bacterial membrane, thereby promoting membrane fusion. This interaction facilitates a more effective intracellular action, resulting in higher local concentrations of the active compounds at the site of action. In addition, liposomal encapsulation provides protection to the incorporated compounds against chemical and enzymatic degradation, contributing to the preservation of their antibacterial efficacy [106]. Moreover, liposomal encapsulation can enhance the biological efficacy of incorporated flavonoids, particularly their antioxidant activity, which has been shown to protect HaCaT keratinocytes against oxidative stress and to support the maintenance of cellular viability [154].

The biosafety of products intended for skin administration is of major importance, in accordance with Food and Drug Administration (FDA) recommendations, due to their direct contact with the skin [135]. Demonstrating the absence of cytotoxicity toward keratinocytes is essential, as these cells constitute the predominant cellular population of the epidermis and play a key role in maintaining skin barrier integrity and regulating epidermal permeability. Therefore, damage to keratinocytes may compromise barrier function and promote inflammatory, irritative, or allergic responses, given their key role in the cutaneous immune response and their ability to secrete cytokines and other inflammatory mediators [155].

Liposomes are generally considered safe and non-toxic. However, in vitro, liposomes have been found to have different effects on evaluated cell lines [156]. Despite this, evidence from the scientific literature indicates that liposomal formulations can enhance the penetration of incorporated substances, increase the cutaneous tolerability of topically applied compounds, protect the encapsulated compounds from degradation, and reduce both systemic and local skin toxicity, thereby improving their overall tolerability following topical administration. Moreover, by enhancing therapeutic performance, liposomal carriers may allow for the use of lower effective doses [157,158].

Taken together, according to the obtained results, the encapsulation of *A. clematitis* extract in liposomes, based on both phosphatidylcholine and phosphatidylserine, represents a promising strategy for increasing the biosafety of this natural extract.

Our results demonstrate that the liposomal formulation exhibits antimicrobial activity while maintaining acceptable cellular tolerance in vitro. This suggests potential advantages over existing proprietary extracts or topical formulations and provides a basis for future dermatological studies. Liposomal encapsulation may improve stability, bioavailability, and interaction with microbial or skin cells, consistent with reports of flavonoid-rich plant extracts in topical delivery systems.

## 4. Limitations

Although this in vitro study provided evidence on the biosafety of the samples of interest, there are certain limitations that need to be addressed in future studies. The current evaluation of the samples was conducted using only 2D cell models, which are a reliable aid in understanding biological processes. However, these models carry limitations as they lose essential features, i.e., cell differentiation in the epidermis, due to differences from the in vivo environment. Instead, 3D culture models appeared as [159]. Another aspect that can be listed as a limitation is the treatment time, which in this case was 24 h. Hence, future research studies could focus on longer exposure periods (48 or 72 h) to identify the long-term effects.

## 5. Conclusions

Once the AC extract was obtained by maceration, it was evaluated in terms of its chemical composition. HPLC-MS analysis revealed a total flavonoid content of 67.23 ± 0.33 mg QE/g DW and a polyphenol content expressed as gallic acid of 64.38 ± 0.16 mg GAE/g DW. Based on the antioxidant capacity assessments, the extract showed notable activity, expressed as DPPH − IC_50_ = 0.1619 mg/mL extract and ABTS − IC_50_ = 205.57 μg/mL extract.

The dried extract was encapsulated into four liposomal formulations based on PSA and PCA, which were characterized using Atomic Force Microscopy (AFM), Dynamic Light Scattering (DLS), ZP measurements, and the polydispersity index. From the entrapment efficiency analysis, we can state that the release of encapsulated flavonoids occurs gradually, with the highest amount being released within the first 8 h, exceeding 88% in PSA liposomes.

The biological activity was evaluated through antimicrobial testing and anti-inflammatory assessment using HaCaT skin-derived cells. The extract demonstrated good antimicrobial activity against *S. aureus*, *S. pneumoniae*, *E. coli*, *P. aeruginosa*, and *C. albicans*, indicating potential efficacy in the treatment of various infections associated with skin lesions.

Evaluation in HaCaT skin-derived cells (at 10–100 µg/mL) showed that the samples exhibited overall good tolerability, with only a slight decrease in cell viability (the most statistically significant being associated with AC treatment), and no morphological, nuclear, or mitochondrial structural alterations were observed.

Therefore, due to its complex chemical composition, rich in polyphenols and flavonoids, *Aristolochia clematitis* L. represents a valuable source of bioactive compounds with antioxidant and anti-inflammatory potential. This creates a promising direction for future research aimed at maximizing the therapeutic value of active principles derived from spontaneous flora and beyond, to develop products applicable to the management of dermatological conditions.

However, the results indicated that the incorporation of *A. clematitis* extract into phosphatidylcholine and phosphatidylserine liposomal carriers improves its skin tolerability and biosafety, thereby supporting the potential future use of these species-based formulations in topical applications.

## Figures and Tables

**Table 1:** Composition of liposomes with and without AC extract.

Liposome Type	AC Dry Extract Quantity (mg)	PS Quantity (mg)	PC Quantity (mg)	CHL Quantity (mg)	CNA Quantity (mg)
PSA	100	80	-	2.5	20
PCA	100	-	80	2.5	20
PSE	-	80	-	2.5	20
PCE	-	-	80	2.5	20

**Table 2:** Concentration of the polyphenolic compounds and flavonoids present in the AC extract was determined by HPLC-MS analysis.

No on Chromatogram	Compound	Concentration (μg/mL Extract)
1	P-cumaric acid	6.660 ± 0.399
2	Ferulic acid	12.945 ± 0.388
3	Izoquercitrine	35.837 ± 3.225
4	Rutoside	8.840 ± 0.531
5	Epicatechin	0.235 ± 0.007
6	Protocatechuic acid	1.096 ± 0.054
7	Vanilic acid	0.639 ± 0.051
8	Epigalocatechingalat (EGCG)	1.017 ± 0.061

**Table 3:** Entrapment efficiency of the AC extract in liposomes.

Liposome Type	EE (%)		
	1 Day	15 Days	30 Days
PCA	82.68 ± 2.12	82.51 ± 2.54	81.88 ± 2.70
PSA	84.33 ± 3.42	84.19 ± 2.67	83.75 ± 3.61

Legend: PCA—liposomes containing AC extract and phosphatidylcholine, PSA—liposomes containing AC extract and phosphatidylserine, EE%—entrapment efficiency.

**Table 4:** The percentage of flavonoids released from the two liposomal formulas synthesized as a function of time and the amount trapped in each liposome.

Liposome Type	Pfl_rel/h (%)											
	0.5	1	2	3	4	5	6	7	8	12	24	48
**PSA**	6.11 ± 0.11	15.23 ± 0.25	24.68 ± 0.37	37.73 ± 0.44	47.65 ± 0.14	57.86 ± 0.16	68.32 ± 0.26	80.27 ± 0.53	88.25 ± 0.67	88.56 ± 0.27	89.33 ± 0.54	89.65 ± 0.61
**PCA**	4.96 ± 0.19	11.33 ± 0.36	21.11 ± 0.32	33.3 ± 0.21	43.34 ± 0.64	52.23 ± 0.71	66.12 ± 0.26	76.48 ± 0.41	83.48 ± 0.33	86.14 ± 0.63	87.92 ± 0.13	88.28 ± 0.18

Legend: Pfl_rel/h (%)—percentage of flavonoids released from liposomes expressed as a function of time, PCA—liposomes with AC extract and phosphatidylcholine, PSA—liposomes with AC extract and phosphatidylserine.

**Table 5:** Values obtained from AFM analysis for the 4 types of liposomes.

Sample	Day	Radiation Zone (µm^2^)	Sa (nm)	Sq (nm)	Sp (nm)	Sv (nm)	Sy (nm)
PSA	d1	1088.402 ±12.923	39.1817 ±2.2301	48.6596 ±2.1247	152.552 ±4.022	−115.093 ±8.753	267.645 ±12.452
	d15	1088.927 ±13.125	39.2225 ±3.4001	50.0526 ±2.1777	154.579 ±6.431	−114.341 ±8.113	268.920 ±11.679
	d30	1089.546 ±13.118	40.1112 ±2.8992	41.8991 ±3.1872	157.005 ±5.661	−112.272 ±7.558	269.277 ±12.909
PCA	d1	1116.611 ±9.887	58.2245 ±3.1180	71.0012 ±3.8874	151.200 ±5.471	−150.492 ±6.021	301.692 ±10.842
	d15	1116.440 ±10.001	59.8882 ±4.4440	71.9991 ±6.2282	152.888 ±8.004	−150.004 ±8.337	302.892 ±12.220
	d30	1120.873 ±11.165	63.0074 ±6.2728	73.5516 ±5.0078	155.311 ±7.910	−149.102 ±7.699	304.413 ±11.883
PSE	d1	911.611 ±10.129	13.8296 ±0.2547	17.2035 ±0.5213	41.320 ±0.222	−38.328 ±0.578	79.648 ±0.264
	d15	913.351 ±9.100	14.9942 ±1.6471	20.7413 ±1.8631	44.788 ±0.075	−37.333 ±3.811	82.121 ±3.009
	d30	914.189 ±11.107	16.0461 ±1.0083	22.2456 ±2.0052	46.293 ±3.008	−35.887 ±5.003	82.180 ±5.117
PCE	d1	934.179 ±12.444	19.4207 ±2.6450	24.1761 ±1.8217	60.159 ±5.194	−54.025 ±3.893	114.184 ±7.006
	d15	934.887 ±14.001	19.8971 ±1.8111	24.9199 ±2.1132	61.324 ±4.620	−55.001 ±4.576	116.325 ±7.659
	d30	935.337 ±11.898	21.0241 ±1.6792	26.0045 ±1.8054	63.738 ±4.297	−55.110 ±5.081	118.848 ±9.003

**Table 6:** Zeta Potential and polydispersion index for synthesized liposomes.

Sample	Zeta Potential (mV)		Polidispersion Index (PDI)	
	Moment 0	After 30 Days	Moment 0	After 30 Days
**PSA**	−8.037 ± 1.399	−8.007 ± 1.145	0.348 ± 0.32	0.483 ± 0.29
**PSE**	−14.400 ± 2.173	−14.025 ± 2.212	0.216 ± 0.10	0.286 ± 0.66
**PCA**	−15.133 ± 2.106	−14.571 ± 1.703	0.301 ± 0.05	0.417 ± 0.72
**PCE**	−18.021 ± 2.214	−17.377 ± 2.271	0.244 ± 0.43	0.278 ± 0.16

Legend: PCA—liposomes with AC extract and phosphatidylcholine, PSA—liposomes with AC extract and phosphatidylserine, PSE—empty liposomes with phosphatidylserine, PCE—empty liposomes with phosphatidylcholine, PDI—polydispersity index.

**Table 7:** Antimicrobial activity of AC extract and four synthesized liposomes.

No.	Microorganism	Extract de AC 100 mg/mL	PSA	PCA	PSE	PCE	PEN 10 U	VAN 30 µg	CEF 30 µg	OFL 5 µg	FLC 25 µg	Distilled Water
Zone of inhibition [in mm diameter]												
1	*Staphylococcus aureus* ATCC 25923	16.33	15.00	14.66	NA	NA	30	18	26.33	27	NT	NA
2	*Streptococus pneumoniae* ATCC 49619	12.5	11.33	10.33	NA	NA	26	23	NT	18	NT	NA
3	*Escherichis coli* ATCC 25922	12.66	11.33	11.00	NA	NA	NT	NT	25	30.66	NT	NA
4	*Pseudomonas aeruginosa* ATCC 27853	10.33	9.66	9.66	NA	NA	NT	NT	NT	19	NT	NA
5	*Candida albicans* ATCC 90029	NA	NA	NA	NA	NA	NT	NT	NT	NT	32	NA

AC—AC, PSA—liposomes with phosphatidylserine and AC extract, PCA—liposomes with phosphatidylcholine and AC extract, PSE—liposomes with phosphatidylserine without extract, PCE—liposomes with phosphatidylcholine without extract, PEN—penicillin, VAN—vancomycin, CEF—cefoxitin, OFL—ofloxacin, FLC—fluconazole, NA—no activity, NT—not tested.

## Data Availability

The original contributions presented in this study are included in the article/Supplementary Material. Further inquiries can be directed to the corresponding authors.

## Supplementary Materials

The following supporting information can be downloaded at: https://www.mdpi.com/article/10.3390/pharmaceutics18010089/s1, Figure S1: 2D (up) and 3D (down) AFM image of sample; Figure S2: Profile on selected areas for the sample PSA; Figure S3: Profile on selected areas for the sample PCA; Figure S4: Profile on selected areas for the sample PSE; Figure S5: Profile on selected areas for the sample PCE; Figure S6: The Histograms of diameter distribution for PCA liposomes with encapsulated extract from AC (**a**) and for PCE liposomes without encapsulated extract (**b**); Figure S7: The Histograms of diameter distribution for PSA liposomes with encapsulated extract from AC (**a**) and for PSE liposomes without encapsulated extract (**b**); Figure S8: The Inhibition diameters on *Staphylococcus aureus* (**A**), *Streptococcus pneumoniae* (**B**), *Escherichia coli* (**C**), *Pseudomonas aeruginosa* (**D**) of 1-PCA, 2-PSA, 3-PCE, 4-PSE, 5-AC extract.
