# Supplementary material for: Potentiation of the Pharmacological Effects of an Aristolochia clematitis L. Extract by Loading into Liposomes Facilitating Release to HaCaT Cells
Source: Pharmaceutics. 2026 Jan 10;18(1):89. doi: 10.3390/pharmaceutics18010089 (PMC12845346; doi:10.3390/pharmaceutics18010089)
Supplement: Supplementary file 1 [file pharmaceutics-18-00089-s001.zip › pharmaceutics-4044931-supplementary.pdf]

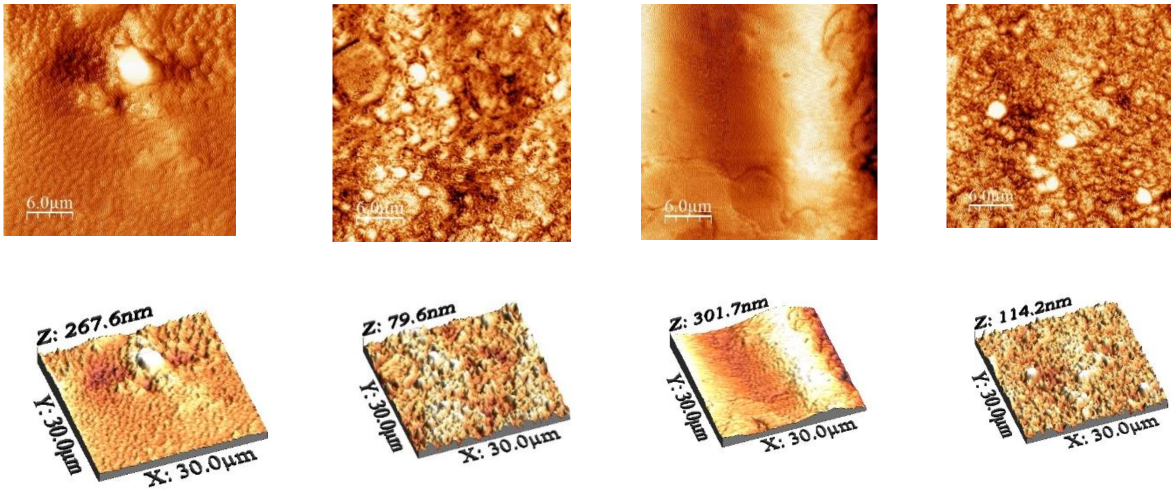

PSA PSE PCA PCE

Figure S1 – 2D (up) and 3D (down) AFM image of sample

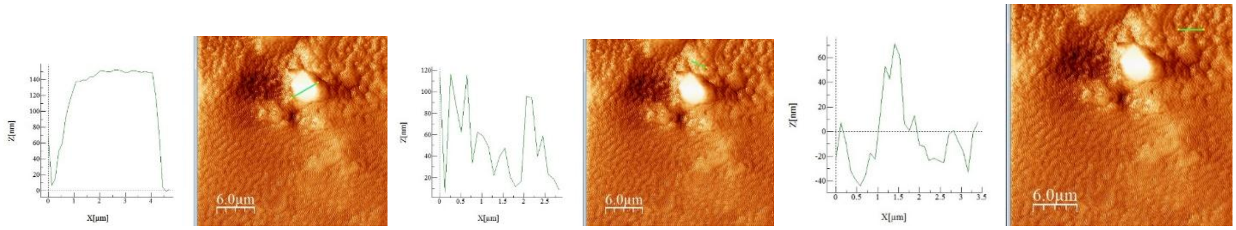

Figure S2 – Profile on selected areas for the sample PSA

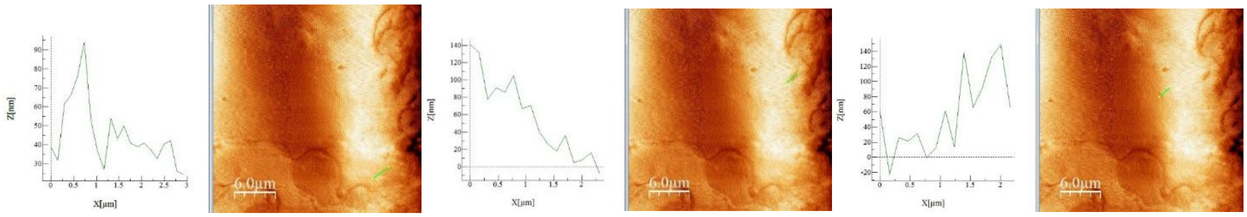

Figure S3 – Profile on selected areas for the sample PCA

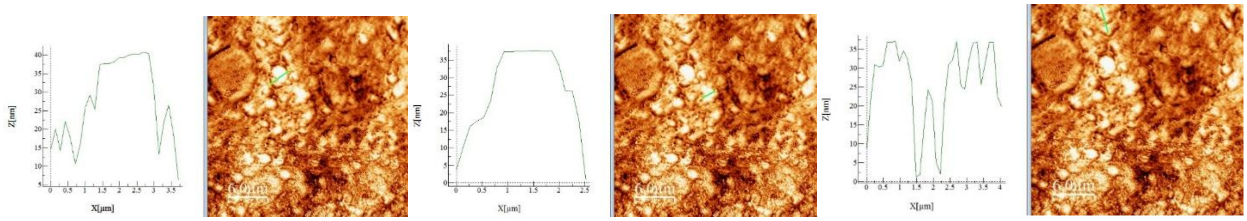

Figure S4 – Profile on selected areas for the sample PSE

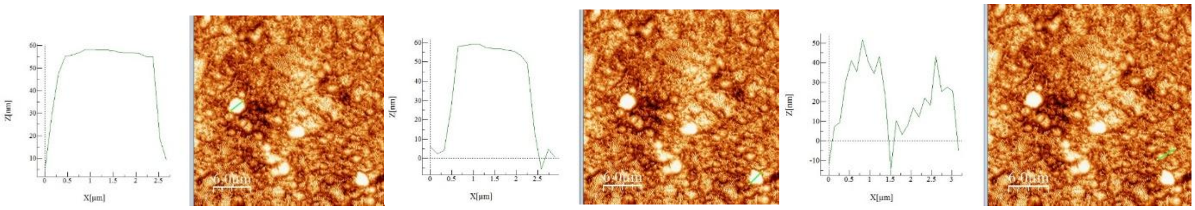

Figure S5 – Profile on selected areas for the sample PCE

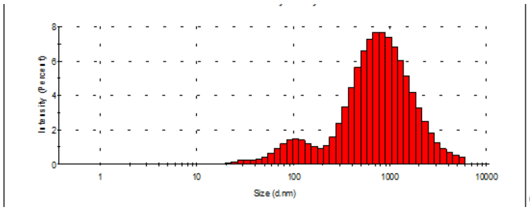

(a)

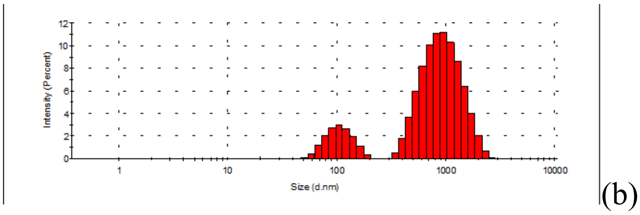

(b)

Figura S6. The Histograms of diameter distribution for PCA liposomes with encapsulated extract from AC (a) and for PCE liposomes without encapsulated extract (b).

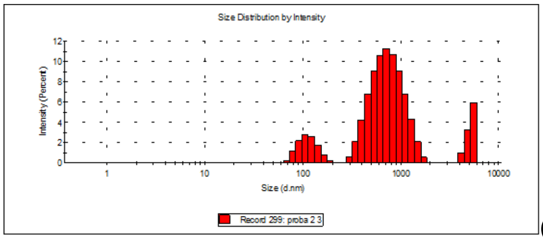

(a)

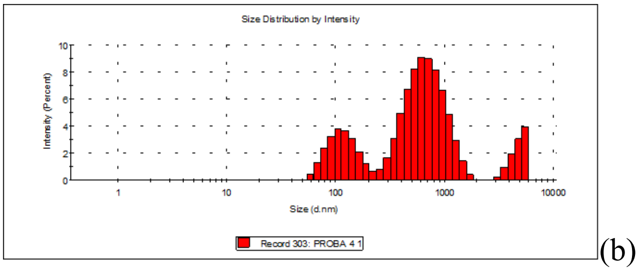

(b)

Figura S7. The Histograms of diameter distribution for PSA liposomes with encapsulated extract from AC (a) and for PSE liposomes without encapsulated extract (b).

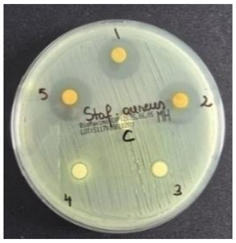

(A)

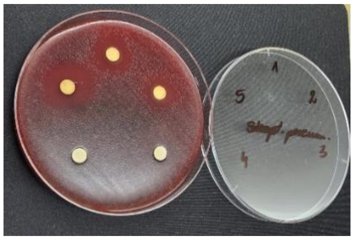

(B)

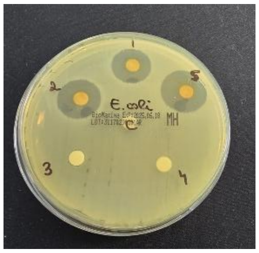

(C)

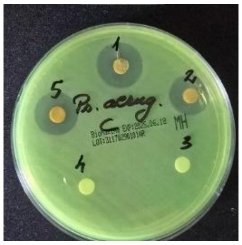

(D)

Figura S8. The Inhibition diameters on *Staphylococcus aureus* (A), *Streptococcus pneumoniae* (B), *Escherichia coli* (C), *Pseudomonas aeruginosa* (D) of 1-PCA, 2-PSA, 3-PCE, 4-PSE, 5-AC extract
